# Supplementary material for: Antibody Tumor Targeting Is Enhanced by CD27 Agonists through Myeloid Recruitment
Source: Cancer Cell. 2017 Dec 11;32(6):777–791.e6. doi: 10.1016/j.ccell.2017.11.001 (PMC5734932; doi:10.1016/j.ccell.2017.11.001)
Supplement: Document S1. Figures S1–S6 [file mmc1.pdf]

Cancer Cell, Volume *32*

Supplemental Information

Antibody Tumor Targeting Is Enhanced by CD27 Agonists through Myeloid Recruitment

Anna H. Turaj, Khiyam Hussain, Kerry L. Cox, Matthew J.J. Rose-Zerilli, James Testa, Lekh N. Dahal, H.T. Claude Chan, Sonya James, Vikki L. Field, Matthew J. Carter, Hyung J. Kim, Jonathan J. West, Lawrence J. Thomas, Li-Zhen He, Tibor Keler, Peter W.M. Johnson, Aymen Al-Shamkhani, Stephen M. Thirdborough, Stephen A. Beers, Mark S. Cragg, Martin J. Glennie, and Sean H. Lim

Supplemental Information

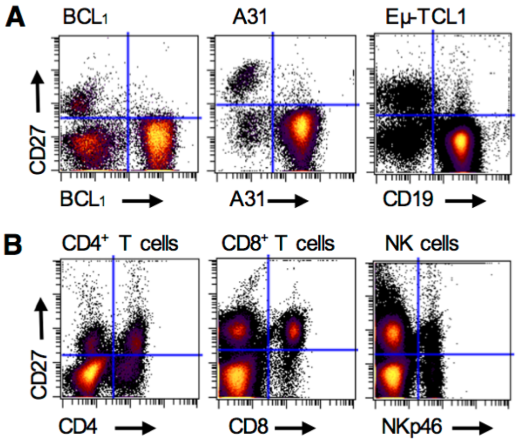

Figure S1. CD27 expression on tumor and immune effector cells. Related to Figure 2.
(A) Expression of CD27 on BCL_1, A31 and Eμ-TCL1 cells purified from mouse splenocytes are shown. Plots are representative of triplicate experiments. (B) Expression of CD27 on $CD4^+$ T cells, $CD8^+$ T cells and NK cells from mouse splenocytes are shown. Plots are representative of triplicate experiments.

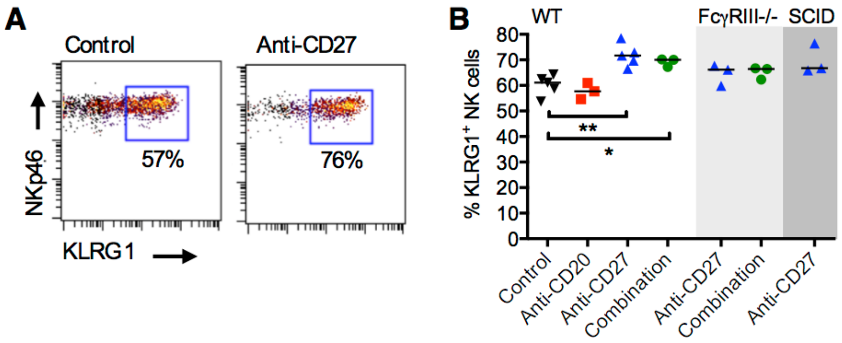

Figure S2. Anti-CD27 activation of NK cells. Related to Figure 3.
(A) Naive mice were treated with isotype control (day 1), anti-CD20 (day 1, 200 µg), anti-CD27 (day 2, 200 µg) or a combination and peripheral blood examined on day 4 for KLRG1 expression on NK cells. Representative plots are shown. **(B)** Graph shows cumulative data from (A), n=3-5. Data were assessed using Student's t test; *$p<0.05$, **$p<0.01$.

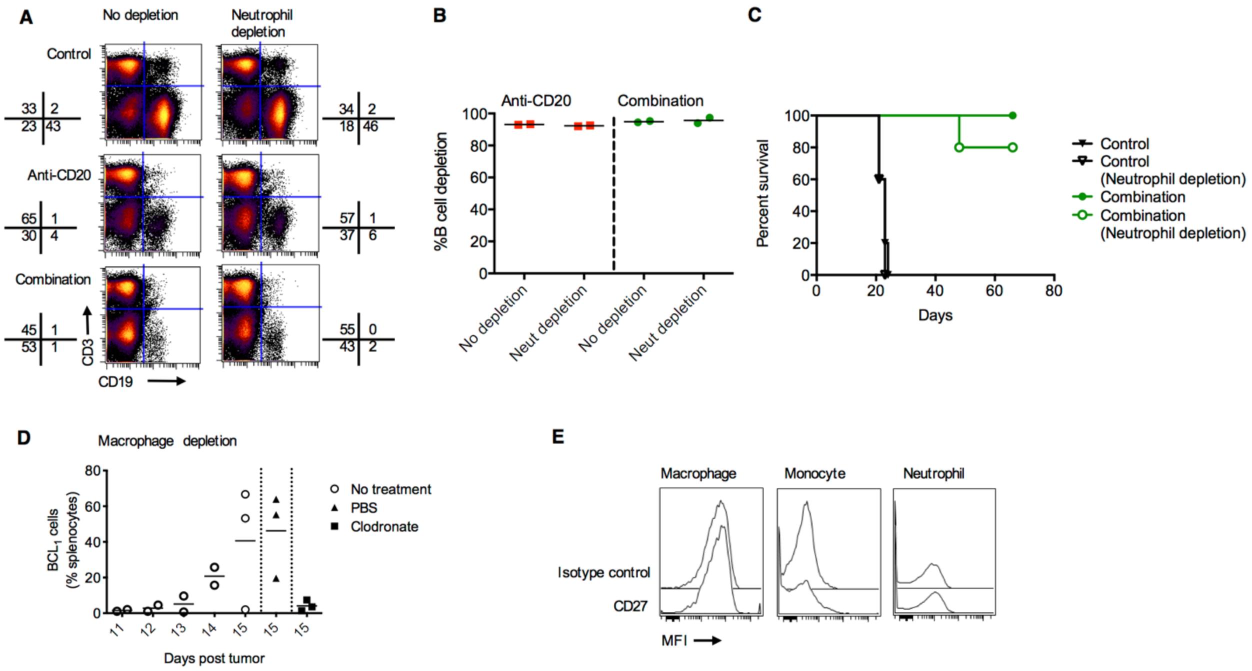

Figure S3. Role of myeloid cells in anti-CD20/CD27 therapy in BCL$_1$. Related to Figure 4.
(A) Naive mice were depleted of neutrophils and then treated with an isotype control, anti-CD20 or anti-CD20/CD27 in combination. Spleens were then harvested and the proportion of B cells in spleens assessed by flow cytometry. Representative plots are shown. (B) Cumulative data from (A), n=2 per group. (C) BCL$_1$-bearing mice were not depleted, or depleted of neutrophils and treated with either isotype control or anti-CD20/CD27 in combination. The Kaplan Meier survival curve is shown. n=9-10 per group, from two independent experiments. (D) Ten thousand BCL$_1$ cells were inoculated i.v. on day 0. Mice were then untreated or treated with PBS or clodronate liposomes on days 11 and 12, to deplete macrophages. The percentage of BCL$_1$ cells in the spleen on days 11, 12, 13, 14 and 15, post tumor inoculation is shown. (E) Expression of CD27 on macrophages, monocytes and neutrophils isolated from splenocytes of BCL$_1$-bearing mice. Histograms are representative of triplicate experiments.

A

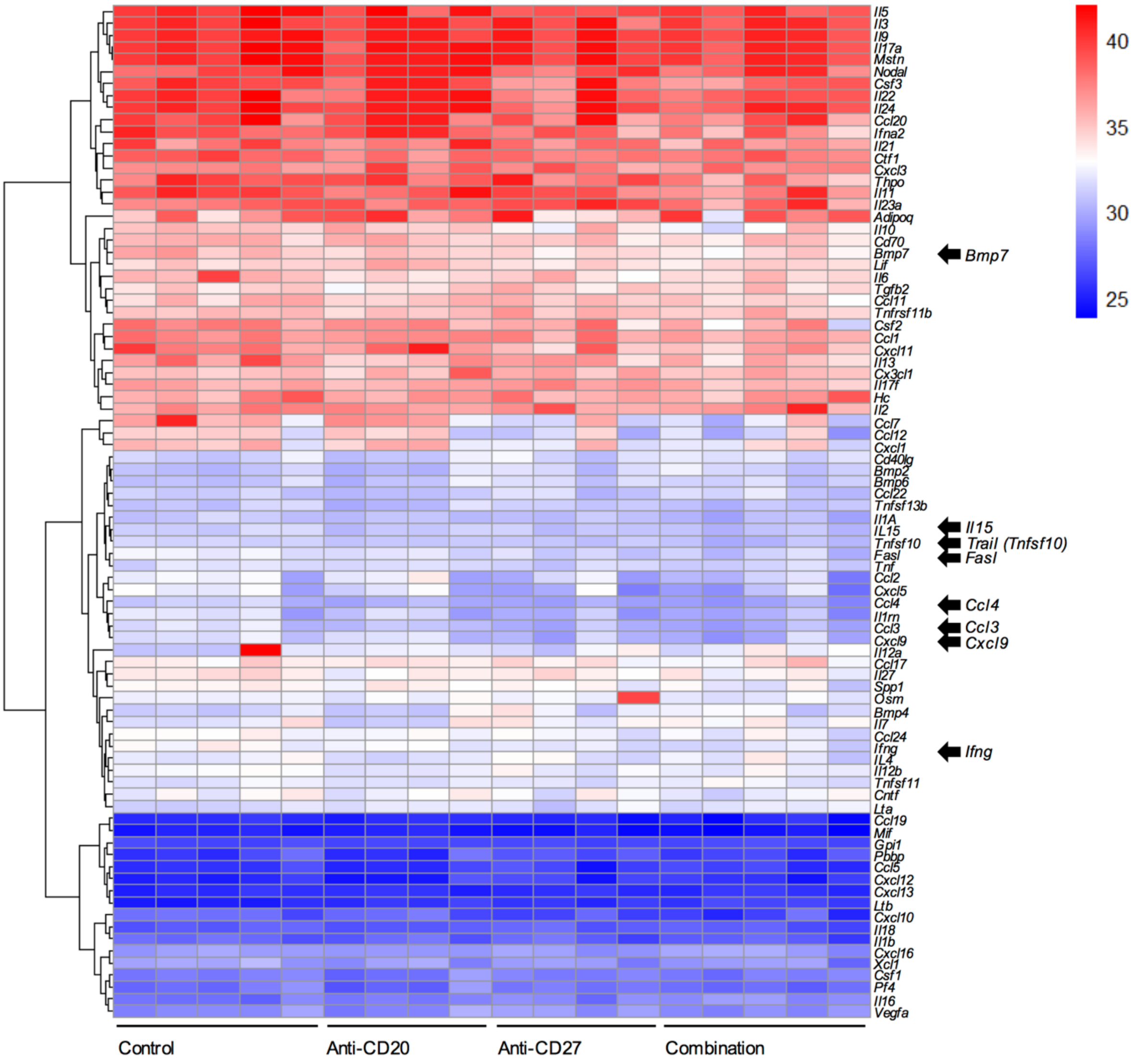
Il5
Il3
Il9
Il17a
Mstn
Nodal
Csf3
Il22
Il24
Ccl20
Ifna2
Il21
Ctf1
Cxcl3
Thpo
Il11
Il23a
Adipoq
Il10
Cd70
Bmp7
Lif
Il6
Tgfb2
Ccl11
Tnfrsf11b
Csf2
Ccl1
Cxcl11
Il13
Cx3cl1
Il17f
Hc
Il2
Ccl7
Ccl12
Cxcl1
Cd40lg
Bmp2
Bmp6
Ccl22
Tnfsf13b
Il1A
IL15
Tnfsf10
Fasl
Tnf
Ccl2
Cxcl5
Ccl4
Il1rn
Ccl3
Cxcl9
Il12a
Ccl17
Il27
Spp1
Osm
Bmp4
Il7
Ccl24
Ifng
IL4
Il12b
Tnfsf11
Cntf
Lta
Ccl19
Mif
Gpi1
Pbbp
Ccl5
Cxcl12
Cxcl13
Ltb
Cxcl10
Il18
Il1b
Cxcl16
Xcl1
Csf1
Pf4
Il16
Vegfa
Bmp7
Il15
Trail (Tnfsf10)
Fasl
Ccl4
Ccl3
Cxcl9
Ifng
40
35
30
25
Control
Anti-CD20
Anti-CD27
Combination

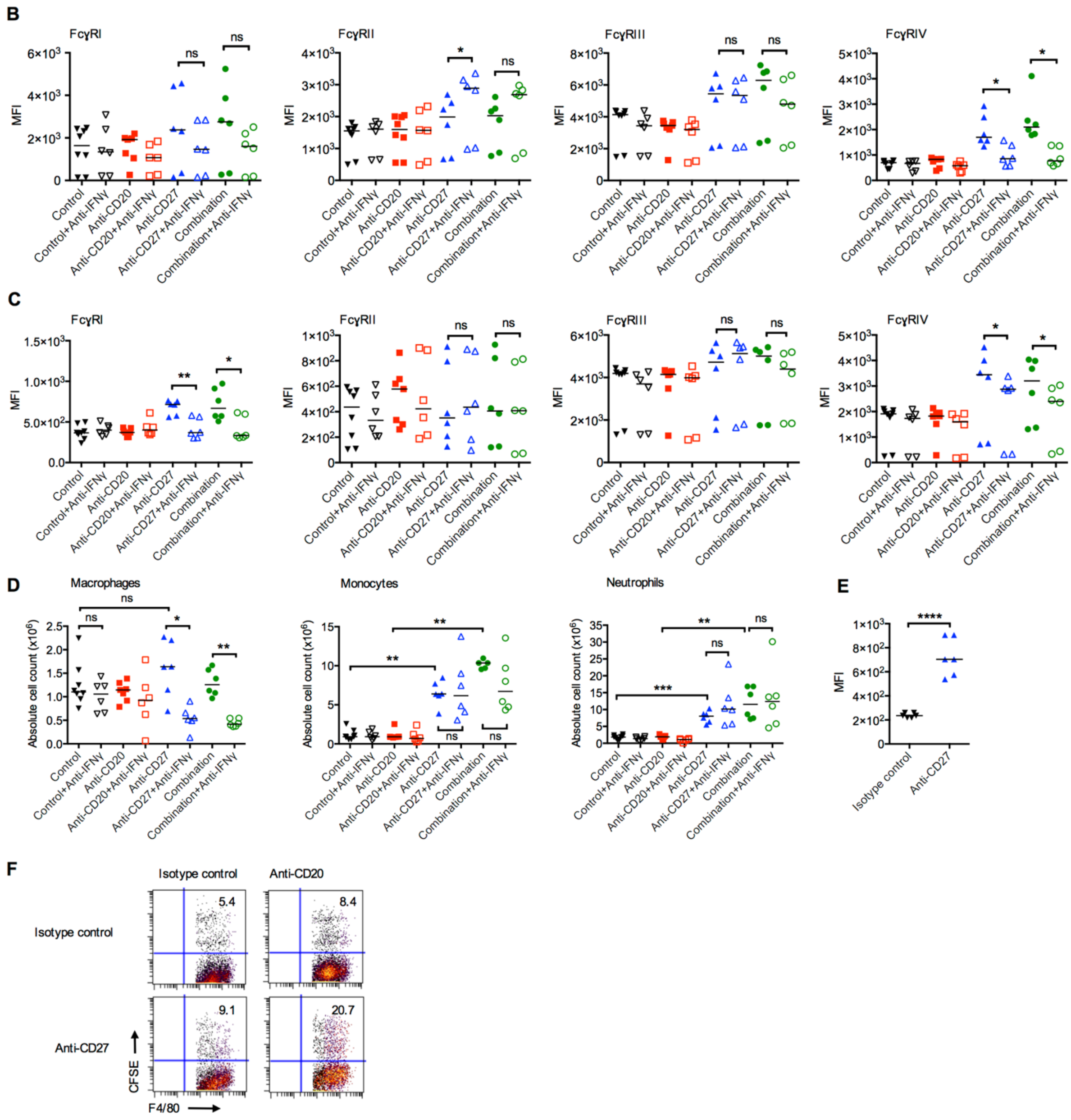

Figure S4. The effect of anti-CD27 on myeloid cells. Related to Figure 5.

(A) Naive mice were treated as described in Figure 2A. Spleens were harvested on day 9 and RNA extracted for cytokine and chemokine profiling. The heatmap shows the normalized CT values for the different treatment conditions and genes tested. (B-C) Tumor-bearing mice were treated with a neutralizing IFNγ as described in Figure 5F. Splenocytes were harvested on day 13 and FcγR expression on monocytes (B) and neutrophils (C) shown. (D) The numbers of macrophages, monocytes and neutrophils from Figure 5F were also enumerated. n=6-8 per group. (E) Naive mice were treated with an isotype control or anti-CD27 and peritoneal macrophages harvested and FcγRIV expression examined. n=6 per group. (F) Representative dot plots of the *ex vivo* phagocytosis assay from Figure 5G are shown. The double positive F4/80$^+$ and CFSE$^+$ cells represent phagocytic cells. The phagocytic index represents fold-increase in phagocytosis over the isotype control, which is normalized to 1. Wilcoxon test was used to assess p values in (B and C) and Student's t test for the remaining data; *p<0.05, **p<0.01, ***p<0.001, ***p<0.0001.

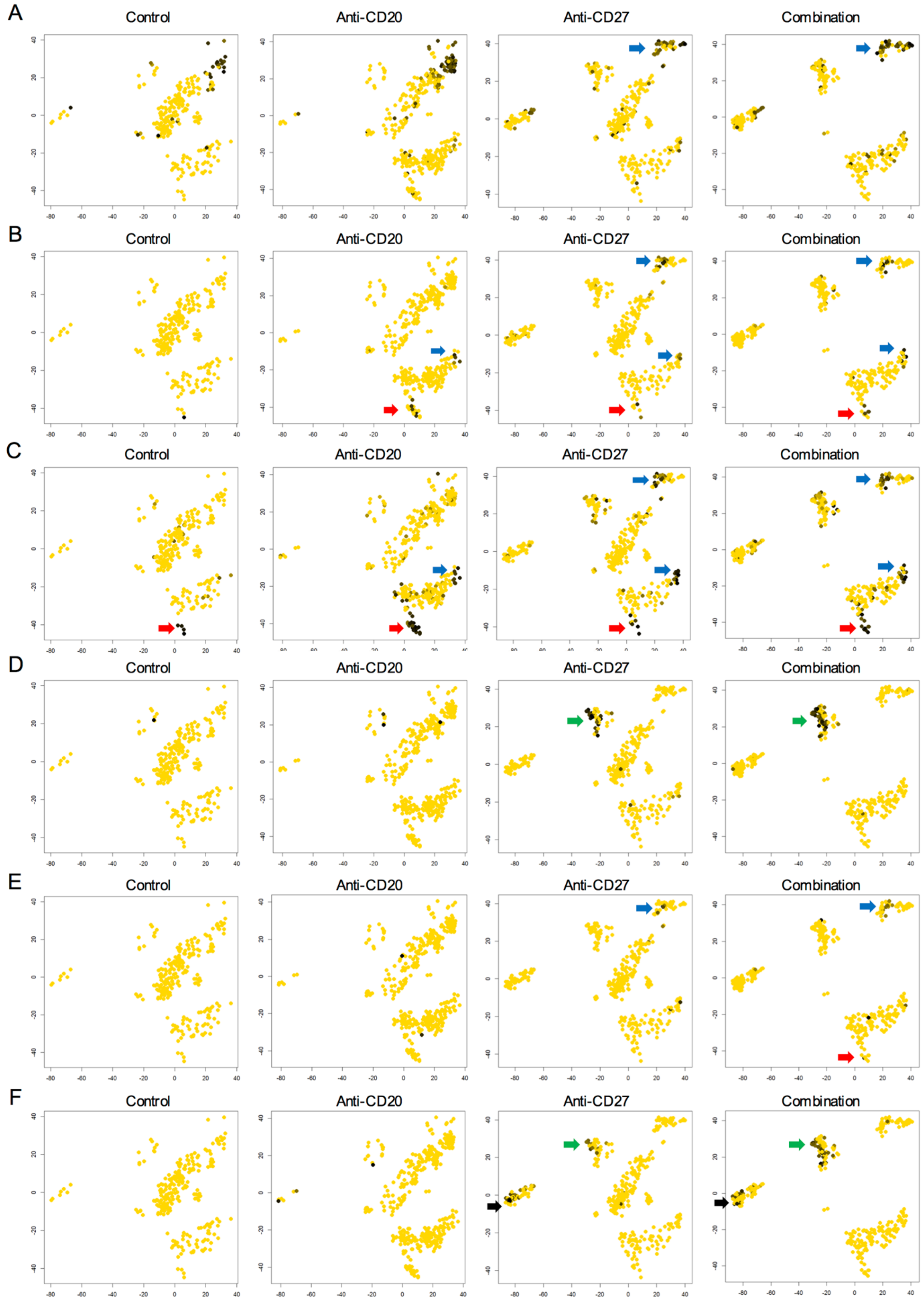

Figure S5. Single cell RNA sequencing from anti-CD20/CD27 therapy. Related to Figure 6. Mice were treated as described in Figure 6. (A) Expression of *Mki67* on effector CD8^{+} T cells are highlighted by the blue arrows. (B) *Ccl4* upregulation on effector CD8^{+} T cells (blue arrows) and NK cells (red arrows). (C)

Ccl5 upregulation is shown on NK cells (red arrows) and effector $CD8^+$ T cells (marked by blue arrows). (D) *Cxcl9* upregulation on macrophages are shown by the green arrows. (E) *Ifng* upregulation was detected on effector $CD8^+$ T cells (blue arrows), and weakly on NK cells (red arrows). (F) *Fcgr4* expression on granulocytes (black arrows) and macrophages (green arrows).

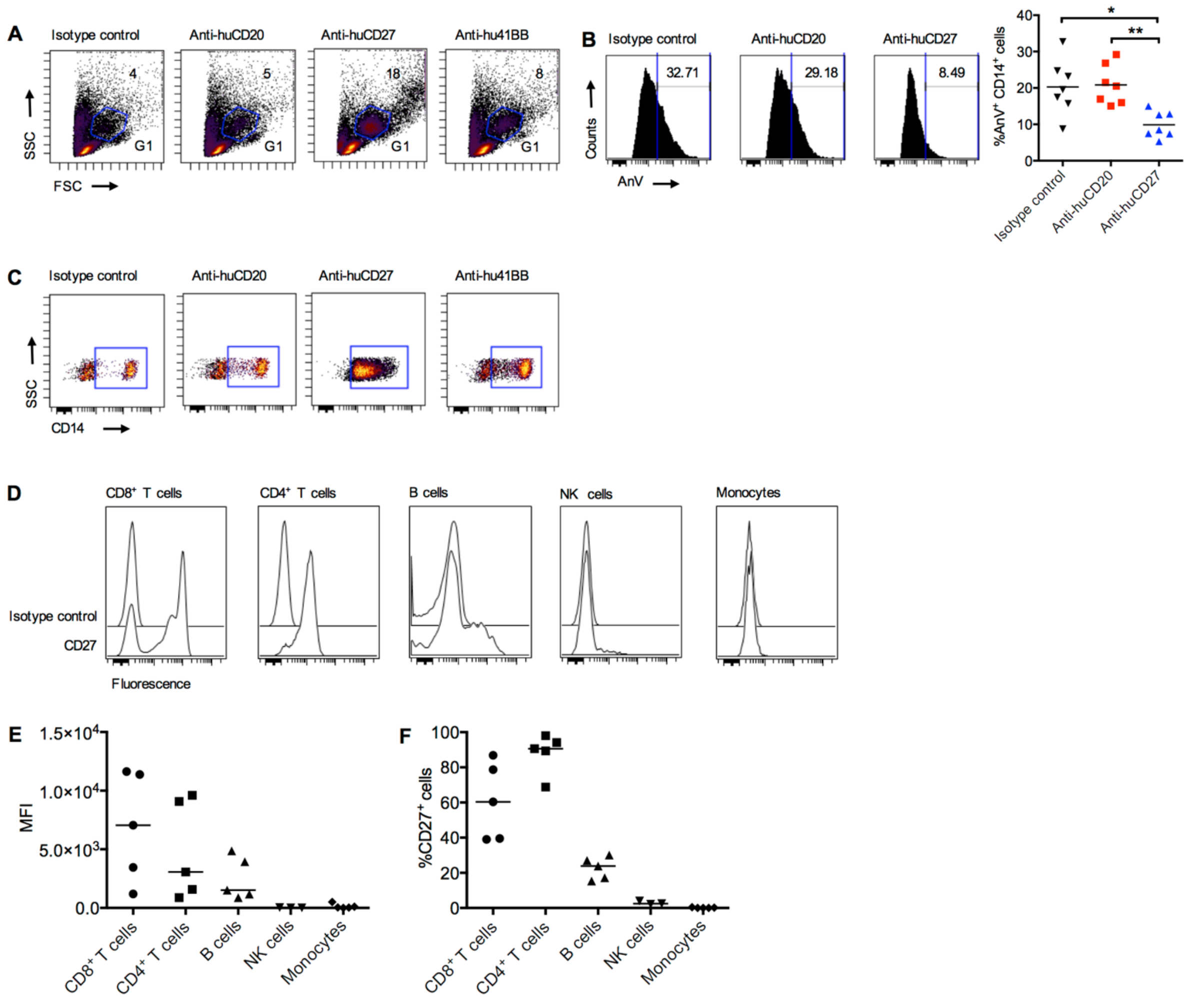

Figure S6. *In vitro* huCD27 stimulation. Related to Figure 7.

(A) Human PBMCs were treated as described in Figure 7N. The gating of monocytes by FSC and SSC are shown (G1). (B) The histograms show percentage of annexin V^+(AnV^+) gated on all $CD14^+$ cells. The cumulative % of AnV^+ $CD14^+$ cells are shown in the graph. n=7. p values were assessed using Student's t test; *$p<0.05$, **$p<0.01$. (C) The level of CD14 expression on G1 gated cells are shown in representative dot plots. (D) Representative histograms showing CD27 expression on $CD8^+$ and $CD4^+$ T cells, B cells, NK cells ($CD56^{dim}$) and monocytes. (E) MFI values from (D) are shown. (F) The % $CD27^+$ cells are shown; n=3-5 donors.
