# Supplementary material for: Antibody Tumor Targeting Is Enhanced by CD27 Agonists through Myeloid Recruitment
Source: Cancer Cell. 2017 Dec 11;32(6):777–791.e6. doi: 10.1016/j.ccell.2017.11.001 (PMC5734932; doi:10.1016/j.ccell.2017.11.001)
Supplement: Document S2. Article plus Supplemental Information [file mmc2.pdf]

Cancer Cell

Article

Antibody Tumor Targeting Is Enhanced by CD27 Agonists through Myeloid Recruitment

Graphical Abstract

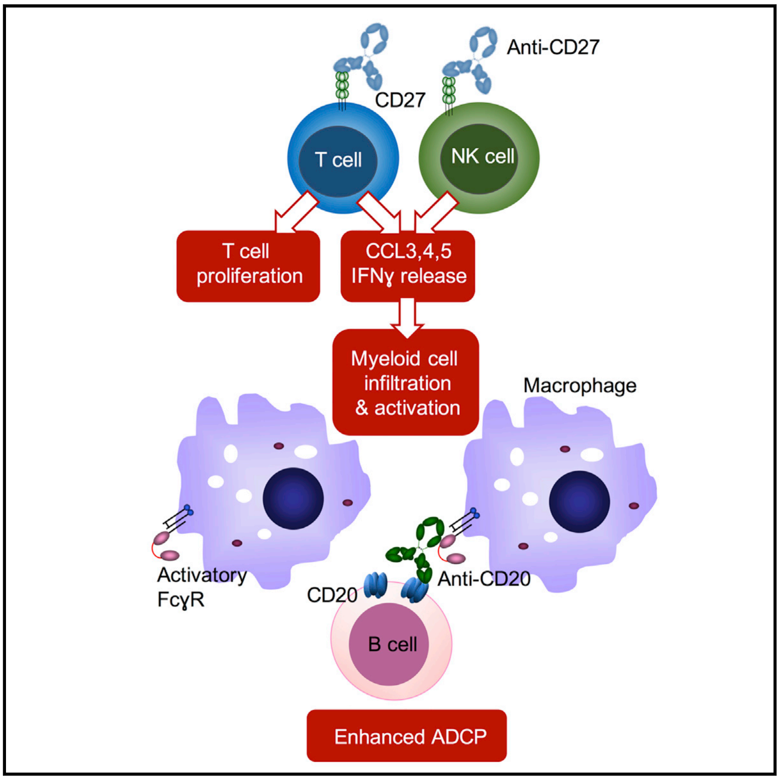

Highlights

- CD27 stimulation enhances anti-tumor efficacy of tumor-targeting mAbs
- Stimulation of CD27 on T and NK cells increases chemokine and IFNγ release
- Released IFNγ leads to myeloid cell activation and infiltration
- Activated macrophages have enhanced anti-CD20 dependent phagocytic capability

Authors

Anna H. Turaj, Khiyam Hussain, Kerry L. Cox, ..., Mark S. Cragg, Martin J. Glennie, Sean H. Lim

Correspondence

s.h.lim@soton.ac.uk

In Brief

Turaj et al. test anti-tumor efficacy of immunomodulatory antibodies combined with anti-CD20 and find that anti-CD27/CD20 has a strong benefit in several tumor models. Anti-CD27 induces IFNγ and chemokines in CD8$^+$ T and NK cells, enhancing macrophage infiltration and activation to promote anti-CD20 activity.

Turaj et al., 2017, Cancer Cell 32, 777–791
December 11, 2017 © 2017 The Authors. Published by Elsevier Inc.
https://doi.org/10.1016/j.ccell.2017.11.001

Cancer Cell
Article

Antibody Tumor Targeting Is Enhanced by CD27 Agonists through Myeloid Recruitment

Anna H. Turaj,[1,2] Khiyam Hussain,[1] Kerry L. Cox,[1] Matthew J.J. Rose-Zerilli,[2] James Testa,[3] Lekh N. Dahal,[1] H.T. Claude Chan,[1] Sonya James,[1] Vikki L. Field,[1] Matthew J. Carter,[1] Hyung J. Kim,[1] Jonathan J. West,[4] Lawrence J. Thomas,[3] Li-Zhen He,[3] Tibor Keler,[3] Peter W.M. Johnson,[2] Aymen Al-Shamkhani,[1] Stephen M. Thirdborough,[2] Stephen A. Beers,[1,2] Mark S. Cragg,[1,2,4] Martin J. Glennie,[1] and Sean H. Lim[1,2,5,*]
[1]Antibody and Vaccine Group, Cancer Sciences Unit, Faculty of Medicine, University of Southampton, Southampton General Hospital, Southampton SO16 6YD, UK
[2]Cancer Research UK Centre, Faculty of Medicine, University of Southampton, Southampton General Hospital, Southampton SO16 6YD, UK
[3]Celldex Therapeutics, Inc., Hampton, NJ 08827, USA
[4]Institute for Life Sciences, Faculty of Medicine, University of Southampton, Southampton SO17 1BJ, UK
[5]Lead Contact
*Correspondence: s.h.lim@soton.ac.uk
https://doi.org/10.1016/j.ccell.2017.11.001

SUMMARY

Monoclonal antibodies (mAbs) can destroy tumors by recruiting effectors such as myeloid cells, or targeting immunomodulatory receptors to promote cytotoxic T cell responses. Here, we examined the therapeutic potential of combining a direct tumor-targeting mAb, anti-CD20, with an extended panel of immunomodulatory mAbs. Only the anti-CD27/CD20 combination provided cures. This was apparent in multiple lymphoma models, including huCD27 transgenic mice using the anti-huCD27, varlilumab. Detailed mechanistic analysis using single-cell RNA sequencing demonstrated that anti-CD27 stimulated CD8^{+} T and natural killer cells to release myeloid chemo-attractants and interferon gamma, to elicit myeloid infiltration and macrophage activation. This study demonstrates the therapeutic advantage of using an immunomodulatory mAb to regulate lymphoid cells, which then recruit and activate myeloid cells for enhanced killing of mAb-opsonized tumors.

INTRODUCTION

Monoclonal antibodies (mAbs) have proven to be potent tools in cancer treatment (reviewed in Weiner, 2015). They can be divided into two groups based on their effector functions: direct tumor-targeting mAbs, such as anti-CD20, anti-Her2, and anti-EGFR target the tumor directly through innate effectors, whereas immunomodulatory mAbs (e.g., anti-PD-1, anti-PD-L1, anti-CTLA-4, and anti-CD40) activate the adaptive immune system. It is generally agreed that direct-targeting mAbs exert their anti-tumor activity by recruiting FcγR-expressing cellular effectors or by blocking oncogenic signaling (Yakes et al., 2002; Gong et al., 2005; Uchida et al., 2004; Beers et al., 2010), whereas immunomodulators either remove inhibitory signaling (checkpoint blockers) or directly stimulate immune effector cells (immunostimulatory mAbs). It has previously been shown that, when combined, certain immunomodulatory mAbs (e.g., 4-1BB) can improve the anti-tumor efficacy of direct tumor-targeting mAbs (CD20) (Souza-Fonseca-Guimaraes et al., 2016) through enhancement of natural killer (NK) cell-mediated antibody-dependent cellular cytotoxicity (ADCC) (Gill et al., 2012; Kohrt et al., 2011), which has moved to the clinical setting for validation. Apart from these data, there are few other studies examining the combination of direct tumor-targeting and immunomodulatory mAbs. To this end, we examined whether the anti-tumor efficacy of the archetypal direct-targeting mAb, anti-CD20, could be enhanced by different immunomodulatory mAbs.

CD27 is a member of the tumor necrosis factor receptor (TNFR) superfamily and exists as a type 1 transmembrane, disulfide-linked homodimer (van Lier et al., 1987). Unlike other TNFR members, which are only expressed following activation, CD27 is constitutively present on all subsets of T cells (van Lier et al.,

Significance

Direct tumor-targeting mAbs kill tumors primarily through macrophage-mediated phagocytosis and have demonstrated activity in numerous different cancers. However, these responses are often partial and transient. We now show that the efficacy of these mAbs can be enhanced by increasing the activity and number of macrophages available to phagocytose opsonized tumor cells. This can be achieved indirectly through stimulation of CD27, a costimulatory receptor expressed constitutively on T and NK cells. Activation of these cells triggers the release of chemokines and cytokines that attract and activate macrophages. We describe the unexpected effects of this immunomodulatory mAb and how it can be harnessed to improve the anti-tumor efficacy of direct tumor-targeting antibodies in multiple tumor types.

© 2017 The Authors. Published by Elsevier Inc.
This is an open access article under the CC BY license (http://creativecommons.org/licenses/by/4.0/).

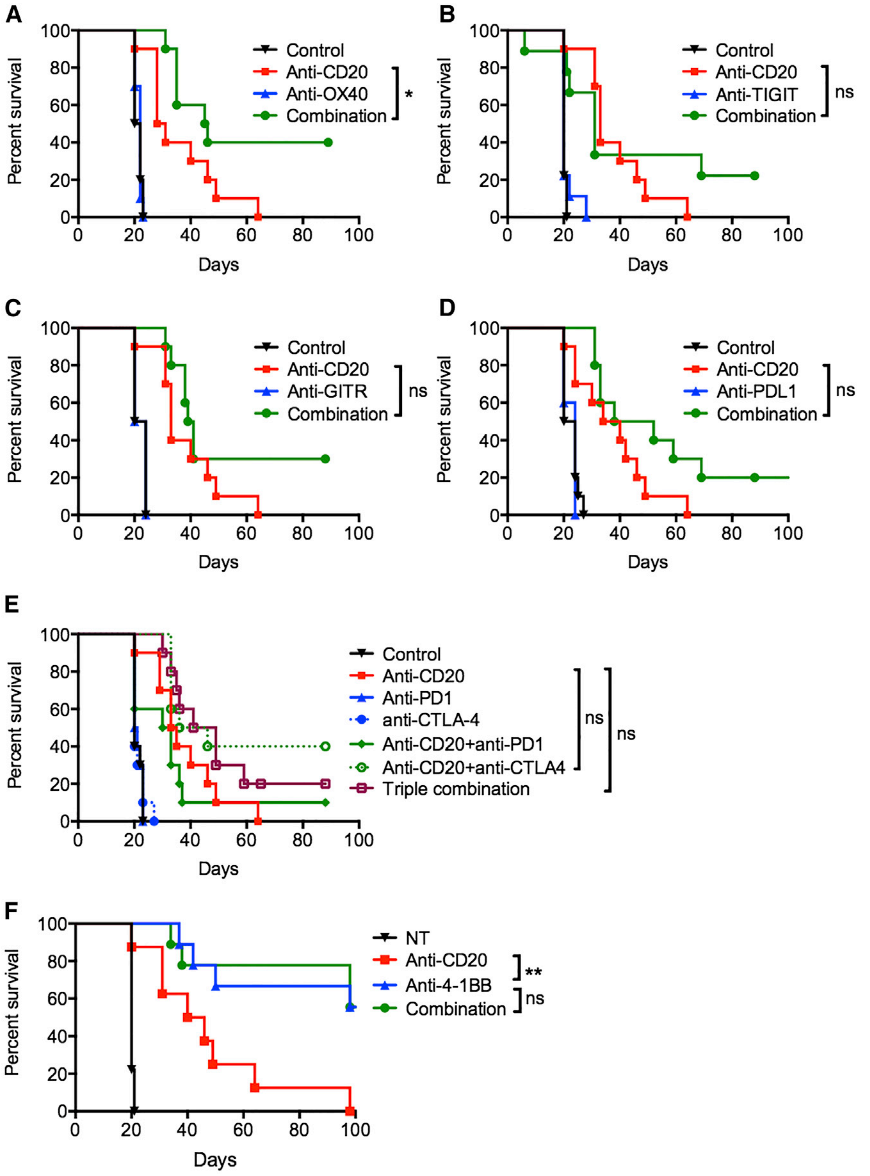

Figure 1. Combining Anti-CD20 with Immunomodulatory mAbs in B Cell Lymphoma

BCL_1-bearing mice were treated with isotype control, anti-CD20 on day 4 (200 μg) alone, or with: (A) anti-OX40 (250 μg) on days 5 and 9; (B) anti-TIGIT (200 μg) on days 5, 8, and 11; (C) anti-GITR (250 μg) on days 5 and 8; (D) anti-PD-L1 (200 μg) on days 5, 7, 9, 11, and 13; (E) anti-PD-1 (250 μg) on days 5, 7, and 9, or anti-CTLA-4 (100 μg) on days 5, 7, and 9; (F) anti-4-1BB (200 μg) on days 5, 8, and 11. Graphs show n = 10 per group, compiled from two independent experiments. Log-rank test was used to assess p values; $^*p < 0.05$, $^{**}p < 0.01$.

1987), a subset of NK cells (Sugita et al., 1992), and memory B cells (Xiao et al., 2004). Upon T cell activation, CD27 expression is further transiently upregulated (de Jong et al., 1991). On T cells, the simultaneous engagement of the T cell receptor (TCR) and CD27, such as by its ligand, CD70, is required for its costimulatory effects (Arens et al., 2001; Keller et al., 2008). CD70-CD27 interaction leads to recruitment of TNFR-associated factor (TRAF) proteins to the CD27 cytoplasmic tail (Akiba et al., 1998; Gravestein et al., 1998). Subsequent activation of canonical and non-canonical nuclear factor-κB (NF-κB) and c-Jun-N-terminal kinase (JNK) signaling pathways follows to elicit cellular responses (Ramakrishnan et al., 2004) involving $CD8^+$ T cell priming, proliferation, survival, and cytotoxicity (Carr et al., 2006; Rowley and Al-Shamkhani, 2004; Taraban et al., 2006). On other cell types, CD70/CD27 interaction supports B cell expansion in the germinal center (Xiao et al., 2004). A subset of NK cells also express CD27, and here engagement of CD27 has been shown to increase interferon gamma (IFNγ) secretion, albeit without a concomitant enhancement of NK cytotoxicity (Takeda et al., 2000). The anti-tumor effect of CD27 ligation has been demonstrated in murine B cell lymphoma (French et al., 2007) and melanoma models (Roberts et al., 2010), and preliminary results of the phase I studies of the anti-human CD27 mAb, varlilumab demonstrate that it is well tolerated and has anti-tumor efficacy (Burris et al., 2017).

RESULTS

Combining Anti-CD20 with Most Immunomodulatory mAbs Does Not Result in Robust Improvements in Therapeutic Efficacy

As an initial screen to investigate whether the therapeutic efficacy of anti-CD20 could be enhanced by combination with immunomodulatory mAbs, we used the syngeneic, immunocompetent murine B cell lymphoma model, BCL_1 (Slavin and Strober, 1978). Anti-CD20 was tested in combination with mAbs to costimulatory receptors, OX40, 4-1BB, GITR, or checkpoint blockers TIGIT, PD-L1, PD-1, or CTLA-4 (Figures 1A–1F). Anti-CD20 alone provided a modest survival benefit with a median survival of 29.5 days compared with 21 days with control mice. When anti-CD20 was combined with immunomodulatory reagents, only anti-OX40 provided a modest benefit with a median survival of 45.5 days (Figure 1A), with the other reagents providing small, non-statistically significant improvements in tumor control. 4-1BB mAb was highly effective as a single agent in this model, with 56% of mice surviving beyond 100 days, but addition of anti-CD20 did not improve its therapeutic efficacy (Figure 1F).

Anti-CD27 Enhances Direct Tumor-Targeting mAb Therapy

An agonistic mAb against CD27 was also tested with anti-CD20 in the same model (Figure 2A). Anti-CD27-treated mice had improved survival compared with control or

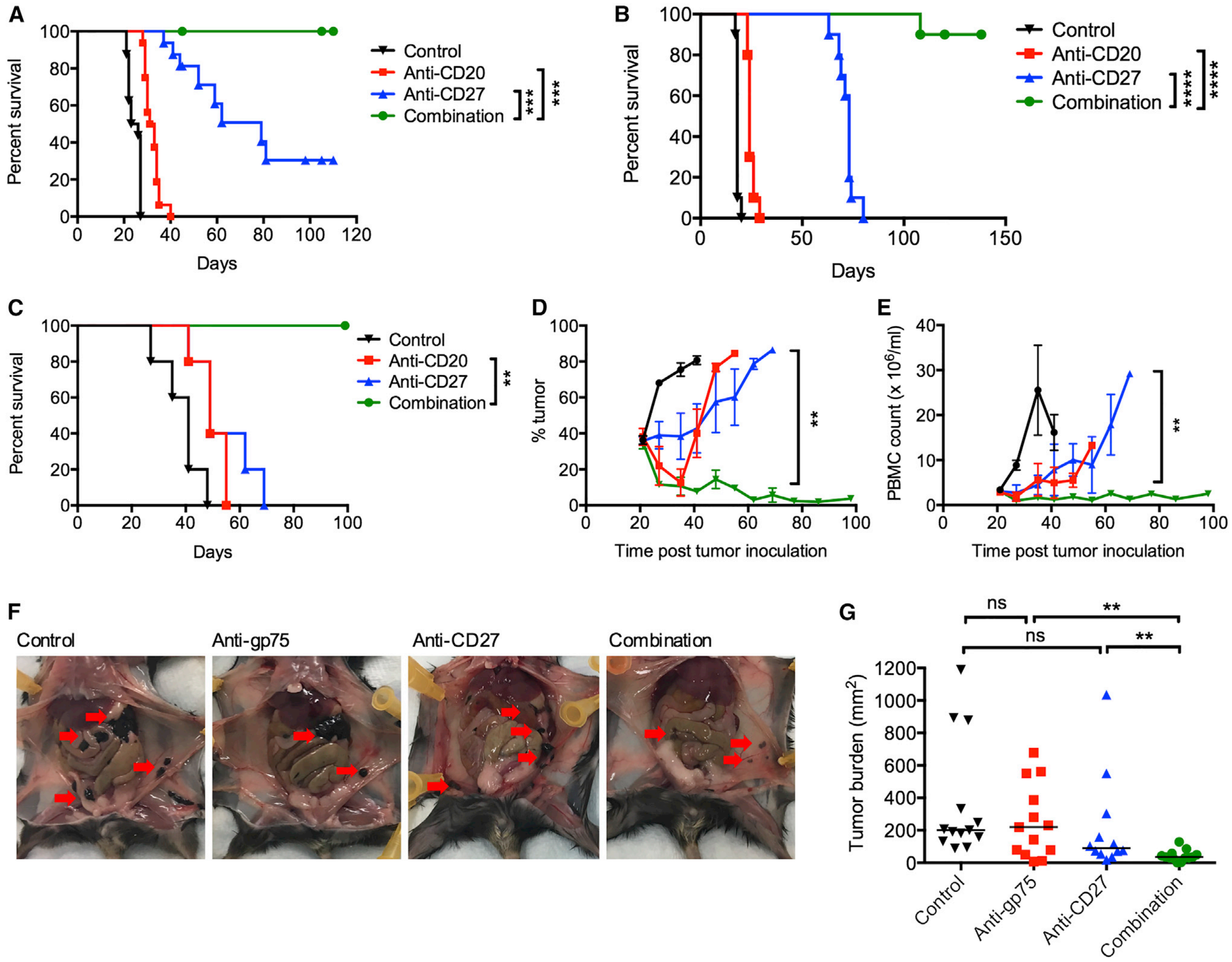

Figure 2. Anti-CD27 in Combination with a Tumor-Targeting mAb

(A) BCL_1-bearing mice were treated with isotype control, anti-CD20 (200 μg) on day 4, anti-CD27 (100 μg) on days 5–8, or in combination. Graph shows n = 15 per group, representative of three independent experiments.

(B) A31-bearing mice were treated with isotype control, anti-CD20 (200 μg) on days 4 and 14, anti-CD27 (100 μg) on days 5–8 and 15–18, or in combination. Graph shows n = 10 per group, compiled from two independent experiments. Data were assessed using log-rank test; ***$p < 0.001$, ****$p < 0.0001$.

(C–E) Eμ-TCL1-bearing mice were treated with anti-CD20 (250 μg) or anti-CD27 (100 μg) the next day, or the combination, >3 weeks post-tumor inoculation when peripheral tumor was >10%. Graphs show n = 6/group, representative of two independent experiments. Animal survival (C), peripheral blood tumor burden (D), and total PBMC count (E). Mean and SEM shown. Log-rank test was used in (C) and Mann-Whitney test in (D and E); **$p < 0.01$.

(F and G) C57BL/6 mice were inoculated with 50,000 B16F10 tumor cells intraperitoneally on day 0, and treated with anti-gp75 (50 μg) on day 0 and anti-CD27 (100 μg) on day 1 (all intraperitoneally). Mice were harvested on day 13, and peritoneal metastases were measured. Representative photographs are shown in (F), with metastases highlighted by the red arrows. The graph in (G) shows data accumulated from two experiments, n = 11–12 mice per group. Mann-Whitney test was used to assess the p value; **$p < 0.01$. See also Figures S1.

anti-CD20-treated mice, but, significantly, when given in combination with anti-CD20, 100% of the mice were cured beyond 100 days.

To ensure that the therapeutic benefit of anti-CD20/CD27 therapy was not confined to the BCL_1 model, the combination was also tested in the A31 B cell lymphoma (Cobb et al., 1986) (Figure 2B) and Eμ-TCL1, B-chronic lymphocytic leukemia (Bichi et al., 2002) models (Figures 2C–2E). In both models, anti-CD20 alone provided a modest therapeutic benefit. Anti-CD27 alone also improved survival of tumor-bearing mice, particularly in the A31 model. However, in combination, a significant improvement was observed, with all mice surviving beyond 100 days. In the Eμ-TCL1 model, therapy provided by either anti-CD20 or anti-CD27 alone was minimal (Figures 2C–2E), yet the combination delivered efficient tumor depletion, leading to experimental cures.

We also examined anti-CD27 with another direct tumor-targeting mAb, anti-gp75, in the B16F10 melanoma model (Figures 2F and 2G), where peritoneal metastases were assessed on day 13 after tumor inoculation (Otten et al., 2008). Anti-gp75 and anti-CD27 individually had some activity in reducing tumor burden but the combination was superior (Figure 2G).

These investigations demonstrate that agonistic anti-CD27 can improve the anti-tumor efficacy of a tumor-targeting mAb to B cell lymphoma in a way not seen with other immunomodulatory mAbs.

Combination Therapy Is Partly Dependent on T Cells

Subsequent investigations aimed to dissect the mechanism by which anti-CD27 enhances anti-CD20 therapy. First, we examined the expression of CD27 on the B cell tumors examined, alongside potential immune effectors (Figures S1A and S1B). Anti-CD27 did not bind to BCL_1, A31, or Eμ-TCL1, and so is not acting as a direct tumor-targeting mAb. Consistent with previous reports (Sugita et al., 1992; van Lier et al., 1987), CD27 was expressed constitutively on NK cells and all subsets of T cells.

Given that CD27 is a well-described costimulatory receptor on T cells, we examined the effect on T cells in the BCL_1 model. BCL_1-bearing mice treated with anti-CD27 and anti-CD20/CD27 had nearly 3-fold more activated $CD8^+$ T cells ($CD62L^{hi}CD44^+$ and $CD62L^{lo}CD44^+$) in the spleen than control and anti-CD20-treated mice on day 13 (Figures 3A and 3B). Further, there was a significant increase in the number of $CD8^+$ T cells in the anti-CD20/CD27 group compared with anti-CD20 alone (median 13.6×10^6 versus 3.6×10^6, respectively) (Figure 3C). A marked increase in $CD8^+$ T cell count was also observed with anti-CD27 alone (median 15.6×10^6), and this was significantly different compared with the control group (median 8.8×10^6). Increased numbers of regulatory T cells (Tregs) were also observed with anti-CD27- and combination-treated groups. Despite the increase in Tregs, the total $CD8^+$/Treg ratio was higher in the combination group compared with anti-CD20 alone (medians 5.9 versus 3.2, respectively).

When the spleens of control and treated BCL_1-bearing animals were examined on days 6 and 9 by immunohistochemistry, a change was observed in the pattern of CD8 staining (Figure 3D). In control and anti-CD20 treated mice, $CD8^+$ staining was confined to the interfollicular regions. In contrast, in anti-CD27- and combination-treated mice, staining was observed in both the interfollicular and follicular regions on day 6 and throughout the whole spleen on day 9. This demonstrates that anti-CD27 given alone, or in combination with anti-CD20, alters the $CD8^+$ T cell distribution.

Altogether, this and published data (French et al., 2007) suggest that anti-CD27's anti-tumor effect is in part mediated by $CD8^+$ T cells. To directly assess their contribution to the immunotherapy in the BCL_1 model, we depleted $CD4^+$, $CD8^+$, or both $CD4^+$ and $CD8^+$ T cells (Figure 3E). As expected, deletion of both $CD4^+$ and $CD8^+$ T cells abrogated anti-CD27 monotherapy. Deletion of either $CD4^+$ or $CD8^+$ alone had minimal impact, but deletion of both $CD4^+$ and $CD8^+$ T cells together reduced therapeutic efficacy of the combination therapy, albeit incompletely. This suggests that, while T cells are important, additional mechanisms of tumor killing compensate for the loss of T cell cytotoxicity in the anti-CD20/CD27-treated cohorts.

The Contribution of NK Cells to Anti-CD20/CD27 Combination Therapy

The other main population of immune effector cells that express CD27 are NK cells, so we explored the ability of anti-CD27 to activate these cells *in vivo*. Naive wild-type (WT), *Fcgr3*$^{-/-}$ (FcγRIII$^{-/-}$) or SCID (severe combined immune deficiency) mice were treated with a single dose of anti-CD27, and the expression of the activation marker, KLRG1, was monitored on peripheral blood NK cells (Figures S2A and S2B). Treatment with anti-CD27 or anti-CD20/CD27 resulted in a ~20% increase in $KLRG1^+$ NK cells compared with controls in WT mice. A similar level of increase was also observed in FcγRIII$^{-/-}$ mice, indicating that NK activation occurred directly via CD27 and not via Fc-FcγR binding (through FcγRIII). Equally, the increase of $KLRG1^+$ NK cells in SCID mice indicates that NK activation does not occur indirectly via CD27-mediated T cell activation as T cells are absent in these mice.

To directly investigate the contribution of NK cells to therapy, they were depleted in the BCL_1 model (Figure 3F) using appropriate doses and formulations of anti-asialo GM1 (Turaj et al., 2017). NK depletion alone did not significantly alter the survival of control or anti-CD20-treated mice. However, there was impairment of survival in the combination-treated mice after NK depletion compared with non-depleted mice. Thus, akin to T cells, anti-CD27 directly activates NK cells, but anti-CD20/CD27 therapy is not entirely dependent on them.

However, when NK and T cells were simultaneously depleted, the therapeutic benefit of adding anti-CD27 to anti-CD20 was abrogated, such that the mice had the same median survival as those treated with anti-CD20 alone (control, 22 days; anti-CD20, 30 days; combination arm with T and NK depletion, 27 days) (Figure 3G). Thus, the therapeutic efficacy of anti-CD20/CD27 therapy requires either T or NK cells to augment tumor control by anti-CD20 by a hitherto unknown mechanism.

Anti-CD27 Promotes Intratumoral Myeloid Cell Infiltration

It is recognized that anti-CD20-mediated antibody-dependent cellular phagocytosis (ADCP) is carried out by myeloid cells (Uchida et al., 2004; Beers et al., 2010). Figures 2D and 2E show that there is a greater level of B cell depletion when anti-CD27 is combined with anti-CD20 in the Eμ-TCL1 model. We sought to validate these findings in the BCL_1 model and to examine whether anti-CD27 altered the myeloid compartment. Spleens of BCL_1-bearing mice were harvested on day 9 or 13 after tumor inoculation, and tumor cells, normal B cells, NK cells, macrophages, monocytes, and neutrophils were enumerated (Figures 4A–4F). Consistent with observations in the Eμ-TCL1 model, anti-CD20 rapidly depleted malignant and normal B cells while minimal difference was seen in the tumor load between control and anti-CD27-treated mice at these time points (Figures 4A and 4B). Combined anti-CD20/CD27 therapy was more effective than anti-CD20 alone in depleting B cells, most evidently with normal B cells at day 9 (means, 12.6×10^6 versus 3.8×10^6, anti-CD20 versus combination) (Figure 4B). We observed a trend toward reduction in splenic NK cells with anti-CD27 and combined anti-CD20/CD27 treatment compared with controls, most noticeably on day 13 (Figure 4C), which is described following NK activation (Robbins et al., 2004).

Examination of the myeloid compartment in the spleen at the same time points demonstrated no significant changes in macrophage numbers (Figure 4D) but marked increases in monocytes and neutrophils (Figures 4E and 4F). There was an

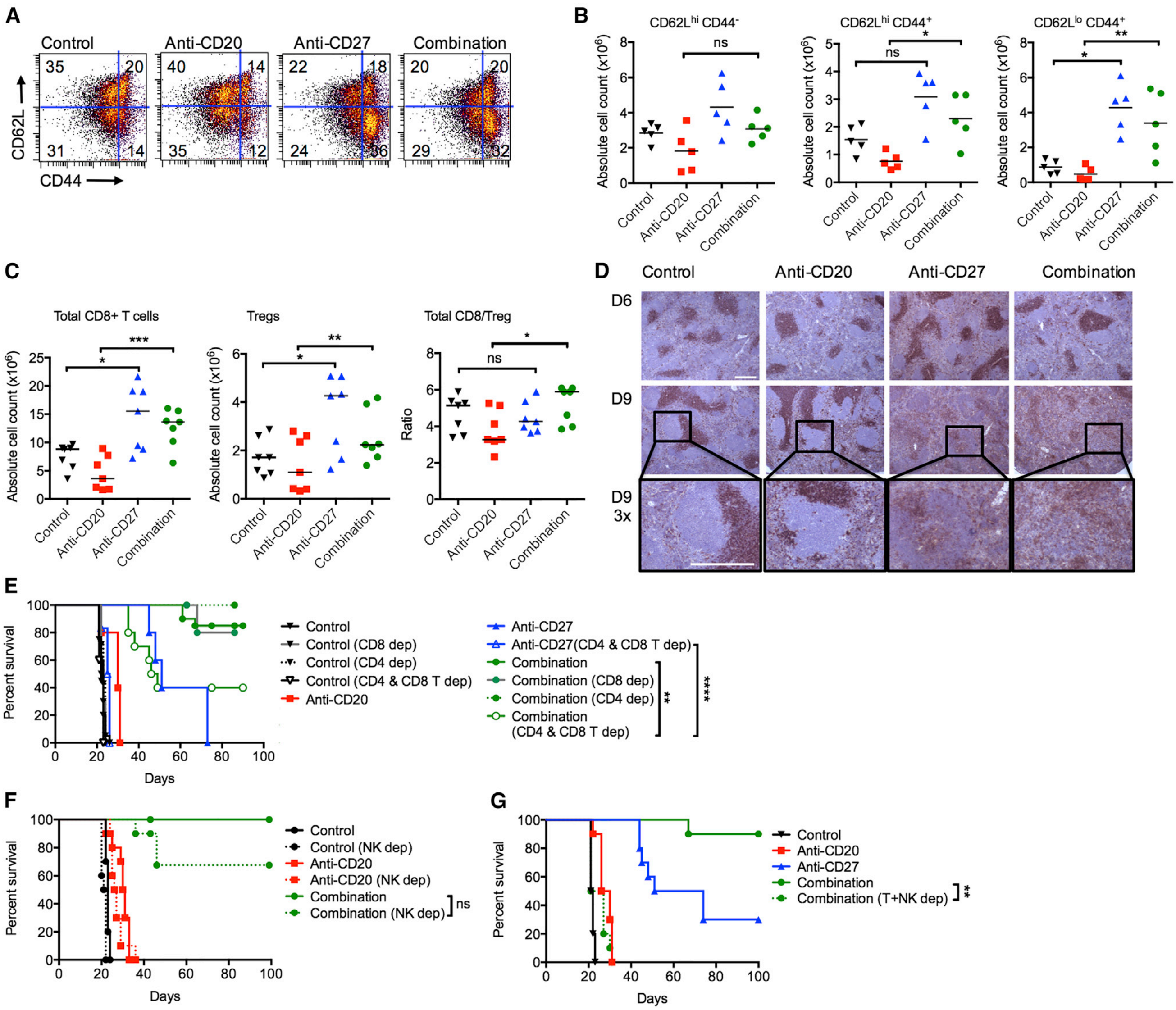

Figure 3. Anti-CD27 Stimulation of CD8$^+$ T cells

(A–C) BCL$_1$-bearing mice were treated as described in Figure 2A, and spleens were harvested on day 13. Cell counts were obtained, and cells were analyzed by flow cytometry. (A) Representative flow cytometry plots showing the percentages of activated CD8$^+$ T cells, denoted by CD62L and CD44 expression, upon treatment with either control, anti-CD20, anti-CD27, or in combination. (B) Graphs showing cumulative data from (A), n = 5 per group, medians shown. (C) Graphs show absolute numbers of CD8$^+$ T cells, Tregs, and CD8$^+$/Treg ratio, n = 7 per group, medians shown, ns indicates not significant. Student's t test was used for (B) and Wilcoxon test for (C) to assess p values; *p < 0.05, **p < 0.01, ***p < 0.001.

(D) BCL$_1$-bearing mice were treated as in (A), and spleens were harvested on days 6 and 9 and stained for CD8 by immunohistochemistry. Both scale bars represent 500 μm. Boxed inset enlarged ×3.

(E) BCL$_1$-bearing mice were depleted of CD8$^+$, CD4$^+$, or both cell types, and treated as described in Figure 2A. Graphs show n = 5–10 per group, representative of two independent experiments, ns indicates not significant.

(F) BCL$_1$-bearing mice were depleted of NK cells and treated with isotype control, anti-CD20, anti-CD27, or the combination. Graphs show n = 5–10, from two independent experiments, ns indicates not significant.

(G) BCL$_1$-bearing mice were depleted of T and NK cells, and treated with isotype control, anti-CD20, anti-CD27, or the combination. Graphs show n = 5, representative of two independent experiments.

Log-rank test was used to assess p values; **p < 0.01, ****p < 0.0001. See also Figure S2.

8- to 9-fold increase in numbers of monocytes and a 5- to 7-fold increase in neutrophils in anti-CD27 and combination arms, compared with control and anti-CD20 alone.

A similar trend of increased myeloid cell infiltration was also observed in the spleen when the experiment was repeated in naive non-tumor-bearing mice (Figures 4G–4I), indicating that these changes are CD27 related and not tumor driven. In this case, macrophage numbers were also significantly elevated in the combination group compared with anti-CD20 alone (means, 2.0 × 10^6 versus 0.7 × 10^6, respectively). Monocytes

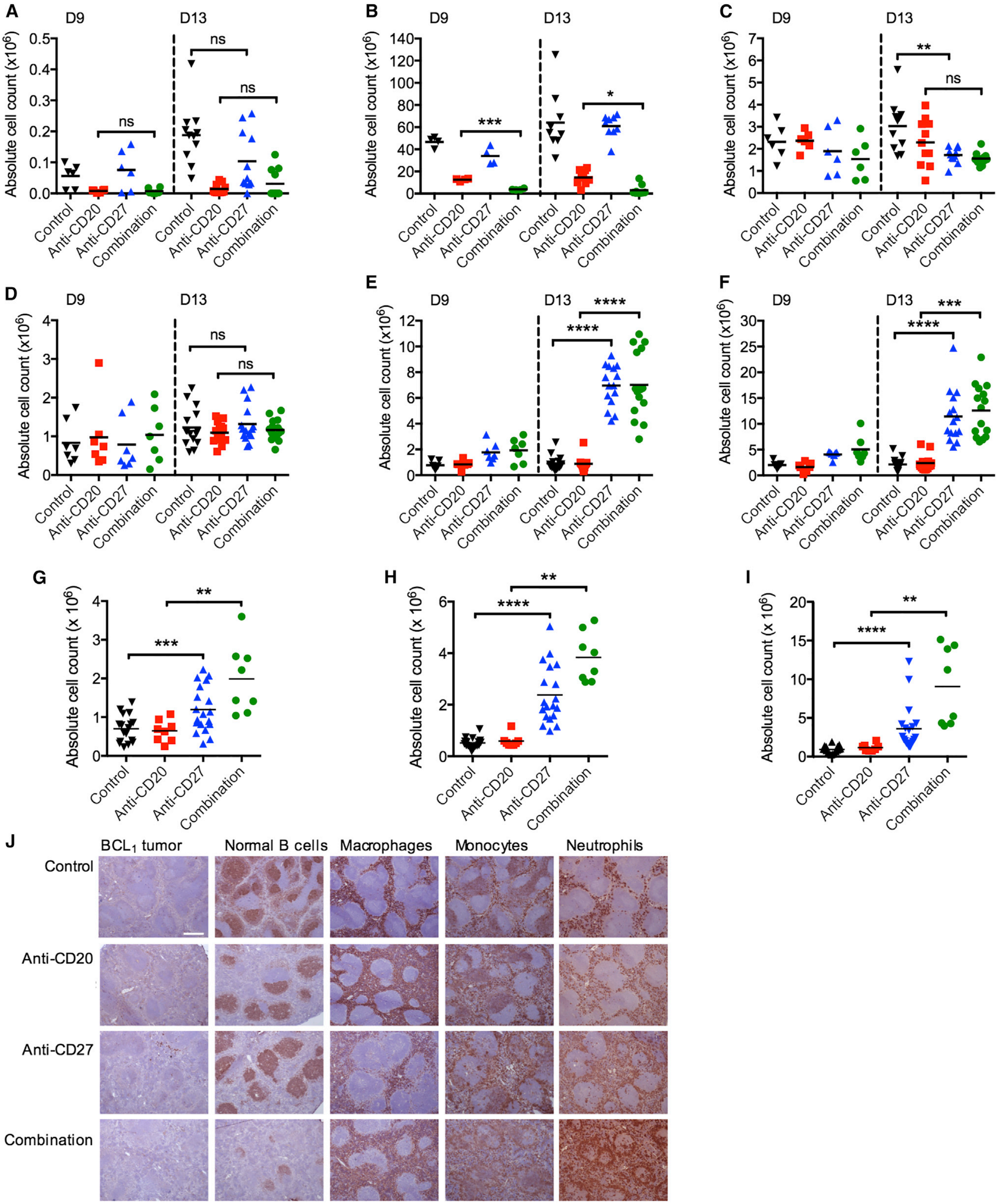

Figure 4. The Effect of Anti-CD27 on Intratumoral Myeloid Cells

(A–F) BCL_1-bearing mice were treated as previously described and spleens harvested on days 9 and 13 and examined for tumor (A), normal B cells (B), NK cells (C), macrophages (D), monocytes (E), and neutrophils (F). Graphs n = 6–15 per group, means shown.

(legend continued on next page)

and neutrophils were increased 6- and 7-fold, respectively (combination group versus anti-CD20 alone).

The spleens of BCL_1-bearing mice were also examined by immunohistochemistry on day 9 (Figure 4J). At this time point, the tumor burden is still low (Figure 4A) and the splenic architecture preserved, allowing changes in immune populations to be better appreciated. Figure 4J shows clear evidence of B cell depletion by anti-CD20 and that this is enhanced by the addition of anti-CD27. Consistent with the flow cytometry data (Figures 4E and 4F), there was increased monocyte and neutrophil staining in the anti-CD27 and anti-CD20/CD27 groups but no obvious increase in macrophages at this early time point.

Depletion of neutrophils had minimal effect on anti-CD20-mediated B cell depletion (Figures S3A and S3B) and there was no statistically significant impact on the survival of anti-CD20/CD27-treated BCL_1 mice (Figure S3C). Macrophage depletion was also performed using liposomal-encapsulated clodronate, but depletion of macrophages itself impairs BCL_1 tumor growth, thus preventing us from assessing the contribution of macrophages to anti-CD20/CD27 therapy (Figure S3D). However, we and others have previously shown that macrophages are the main effectors of anti-CD20-mediated B cell depletion (Minard-Colin et al., 2008; Beers et al., 2010; Tipton et al., 2015b).

Anti-CD27 Indirectly Promotes Infiltration and Activation of Myeloid Cells

CD27 is not expressed on macrophages, monocytes, or neutrophils (Figure S3E), ruling out direct activation of these cells by anti-CD27. We hypothesized that, upon activation by anti-CD27, T and NK cells release chemokines and cytokines that attract and activate myeloid cells. To investigate this, naive mice were treated with anti-CD20, anti-CD27, or the combination, and mRNA was isolated from the spleens and analyzed with a chemokine and cytokine gene expression array (Figure S4A). Genes that were significantly upregulated in anti-CD27 and/or combination arms in comparison with the control group are shown in Figure 5A. We observed increased expression of *Fasl*, *Trail* (*Tnfsf10*), and *Ifng* in mice treated with anti-CD27 ± anti-CD20, supportive of increased T and/or NK cytotoxicity. *Cxcl9*, which is associated with a T_H1 response, and T cell trafficking (Guirnalda et al., 2013; Loetscher et al., 1996) was also increased, in line with the changes observed in Figure 3D. Conversely, no significant change was observed in cytokines associated with T_H2 responses, such as *Il4*, *Il5*, and *Il13* (Figure S4A). Furthermore, chemokines associated with myeloid cell trafficking, such as *Ccl3* and *Ccl4*, were elevated in mice treated with anti-CD20, anti-CD27, and the combination.

It is recognized that macrophages are phenotypically and functionally plastic and have the capacity to both kill tumor and promote its development (Biswas and Mantovani, 2010), with both types capable of mediating ADCP (Dahal et al., 2017). The upregulation of *Ifng* and *Cxcl9* by anti-CD27, and combination therapy (Figure 5A), indicate that these treatments polarize the macrophages toward an anti-tumor phenotype. Consistent with these data, mice treated with anti-CD27 also upregulate CD86 (2-fold) and major histocompatibility complex (MHC) class II (3-fold) on macrophages (Figure 5B). Furthermore, in the BCL_1 tumor microenvironment, anti-CD27 and anti-CD20/CD27 also increased the expression of FcγRIV, a key activatory FcγR involved in mediating ADCP on murine macrophages (Hamaguchi et al., 2006; Biburger et al., 2011; Gul et al., 2014; Tipton et al., 2015a) (Figure 5C). In combination treatment, FcγRIV increased 3-fold compared with anti-CD20-treated mice. No obvious changes were observed in the expression of the other activatory FcγRs, FcγRI, and FcγRIII or the inhibitory FcγR, FcγRII on macrophages. On monocytes, anti-CD27 and combination therapy upregulated all of the FcγRs, but the biggest change was seen with FcγRIV, where there was a 2.5-fold upregulation compared with anti-CD20 (Figure 5C). On neutrophils, the combination arm upregulated all of the activatory FcγRs (FcγRI, FcγRIII, and FcγRIV) but to a lesser degree than on macrophages and monocytes.

To confirm that myeloid cell activation was occurring indirectly through T and NK cells, both populations were depleted in BCL_1-bearing mice treated with anti-CD27 therapy (Figure 5D). In the absence of T and NK cells, all the FcγRs failed to upregulate. Further, the profound infiltration of intratumoral monocytes and neutrophils previously observed (Figures 4E and 4F) was also abrogated (Figure 5E).

We further hypothesized that IFNγ released by anti-CD27-activated T and/or NK cells mediated these myeloid changes (Figure 5F). *In vivo* neutralization of IFNγ in BCL_1-treated mice also abrogated the upregulation of FcγRIV on macrophages and monocytes (Figures 5F and S4B). On neutrophils, the FcγRIV upregulation was attenuated to a lesser extent (Figure S4C). FcγRI upregulation was also reduced on monocytes and neutrophils (Figures S4B and S4C). Finally, the neutralization of IFNγ also reduced the infiltration of macrophages in the combination arm but not monocytes or neutrophils (Figure S4D). Altogether, these experiments demonstrate that the upregulation of FcγRIV on myeloid cells is entirely dependent on IFNγ released by anti-CD27-activated T and/or NK cells, and that IFNγ also contributed toward intratumoral macrophage recruitment.

To demonstrate that anti-CD27-activated macrophages have improved ADCP capacity, peritoneal macrophages treated with anti-CD27 *in vivo* were harvested and examined in an *ex vivo* phagocytosis assay. Like splenic macrophages, peritoneal macrophages upregulated FcγRIV $\sim$3.5-fold following anti-CD27 treatment (Figure S4E). Moreover, anti-CD27-activated macrophages phagocytosed significantly more anti-CD20-opsonized B cells than isotype-control-treated macrophages (5-fold increase in phagocytic index) (Figures 5G and S4F).

(G–I) Naive mice were treated as in (A–F) and spleens harvested on day 13 and examined for macrophages (G), monocytes (H), and neutrophils (I) (n = 8–17 per group), means shown.

Student's t test (A, C–E, and G) and Wilcoxon test (B, F, H, and I) were used to assess p values; *p < 0.05, **p < 0.01, ***p < 0.001, and ****p < 0.0001.

(J) BCL_1-bearing mice treated as in (A–F) and spleens harvested on day 9 and stained for tumor (BCL_1), normal B cells (B220), macrophages (F4/80), monocytes (CD14), and neutrophils (Ly6c/Ly6g) by immunohistochemistry. Scale bar represents 500 μm. See also Figure S3.

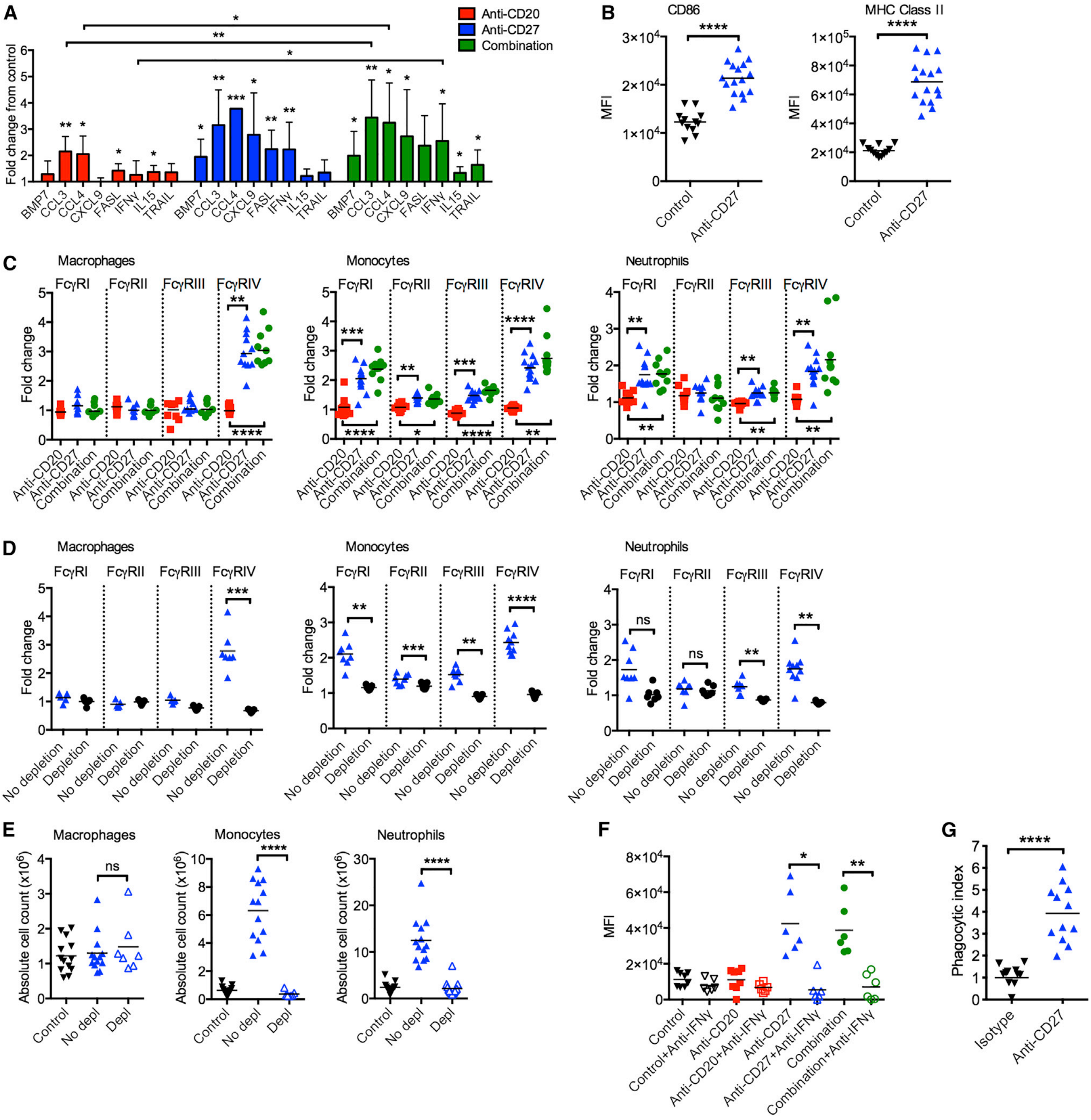

Figure 5. The Effect of Anti-CD27 on Macrophages

(A) Naive mice were treated as described in Figure 2A, spleens were harvested on day 9, and RNA was extracted for cytokine and chemokine profiling. The graph is a summary of the genes that are significantly upregulated over the control sample. Means and 95% confidence interval limits shown.

(B) CD86 and MHC class II expression on splenic macrophages in naive mice treated with isotype control or anti-CD27. MFI values are shown. Means are shown, n = 12–16/group.

(C) BCL_1-bearing mice were treated as previously described, and myeloid cells were harvested on day 13 and FcγR expression assessed. The fold change of MFI values over isotype-treated mice are shown. Means are shown, n = 7–11 per group.

(D) Tumor-bearing mice treated with isotype control or anti-CD27 mAb therapy as previously described were not depleted (No depletion) or simultaneously depleted of T and NK cells (Depletion). Myeloid cells were harvested on day 13, and FcγR expression was assessed. The fold change of MFI values over isotype-treated mice are shown. Means are shown, n = 7–10 per group.

(E) Mice were treated as in (D). Absolute numbers of myeloid cells in the spleen of isotype control, combination treated (No depletion), and combination treated (T and NK depletion) are depicted. Means are shown, n = 7–13 per group.

(legend continued on next page)

Single-Cell RNA Gene Expression Profiling of Anti-CD20/CD27 Therapy

To further characterize cell-type-specific changes elicited by anti-CD27 treatment, single-cell RNA sequencing was performed on cells isolated from spleens of BCL_1-bearing mice (day 13), treated as previously described (Figures 6A–6E and S5A–S5F). We observed a number of changes consistent with the flow cytometry and qPCR array data. First, there was a striking absence of B cells within the combination arm, when compared with anti-CD20 alone (Figure 6B), indicative of enhanced B cell depletion with combination therapy. Second, myeloid and effector $CD8^+$ T cells were markedly increased in the anti-CD27 and combination arms, as illustrated by the higher cell counts (Figure 6B) and expression of the proliferation marker, *Mki67* (Figure S5A) in these populations. Chemokines associated with myeloid cell infiltration, specifically *Ccl3* (Figure 6C), *Ccl4* (Figure S5B), and *Ccl5* (Figure S5C), were upregulated upon anti-CD27 and combination treatment. All three chemokines were primarily expressed in effector $CD8^+$ T cells, with lower levels of expression in NK cells. *Cxcl9* was also upregulated in macrophages with anti-CD27 and combination therapy (Figure S5D). The upregulation of *Ifng* (Figure S5E) observed in the qPCR array was also observed here with anti-CD27 and combination therapy, and seen in effector $CD8^+$ T cells, with some expression in NK cells. Strong *Fcgr4* expression (the gene encoding FcγRIV) was observed in the granulocyte and macrophage populations (Figure S5F), which also displayed upregulation of interferon response genes, *Ifitm3* and *Isg15* (Figures 6D and 6E).

Consistent with the previous results, these data show that anti-CD27 activates $CD8^+$ T cells, and to a lesser extent NK cells, to release CCL3, CCL4, and CCL5, which potentially increases myeloid cell infiltration. Activated $CD8^+$ T cells release IFNγ, which activates myeloid cells, as demonstrated by increased FcγRIV expression.

Anti-huCD27 mAb Displays Similar Activity

To confirm that these findings have relevance for humans, BCL_1 cells were inoculated into human CD27 transgenic (huCD27 tg) mice (Figure 7A). In this model, the clinical candidate anti-huCD27 mAb, varlilumab (Vitale et al., 2012), was employed. As before, we observed a modest improvement in survival with anti-CD20 alone (median survival 44 days). The therapeutic effect of anti-huCD27 monotherapy was again modest, but when given in combination with anti-CD20, significantly enhanced survival benefit was seen with >70% of mice treated with the combination surviving >100 days compared with 0% with either monotherapy.

In keeping with previous data, we observed enhanced B cell depletion (Figures 7B and 7C). The proportion of NK cells was similar for all treatment arms (Figure 7D). An increase in $CD8^+$ T cells or Tregs was not observed with anti-CD27 and combination therapy, but overall, there was an increased $CD8^+$/Treg ratio in the combination arm compared with the other groups (Figures 7E–7G). A modest increase in macrophages was observed here in the combination arm (means 0.55 versus 0.92, anti-CD20 versus combination, respectively) and, similar to the WT mouse CD27 model, there was increased infiltration of monocytes and neutrophils (1.8-fold and 1.6-fold, combination versus anti-CD20) (Figures 7H–7J). Examination of FcγR expression on the myeloid cells also demonstrated upregulation of FcγRIV on macrophages, monocytes, and neutrophils in the combination arm compared with anti-CD20 alone (Figures 7K–7M). Thus, similar to its mouse counterpart, anti-huCD27 is capable of improving anti-CD20-mediated B cell depletion, and this is associated with increased myeloid infiltration and activation.

Next, we studied the *in vitro* effects of anti-huCD27 on peripheral blood mononuclear cells (PBMCs) derived from healthy human donors (Figures 7N–7P and S6A–S6F). PBMCs were incubated with anti-huCD20, anti-huCD27, or T cell-stimulating anti-hu4-1BB for 48 hr before analysis by flow cytometry. Cultures treated with anti-huCD27 contained 3-fold more monocytes than cultures treated with the other mAbs (Figures 7N and S6A) and fewer annexin V^+CD14^+ cells (2-fold less) (Figure S6B), suggesting that anti-huCD27 improved monocyte viability. On further examination, the level of CD14 on monocytes was seen to be downregulated >2-fold after anti-huCD27 treatment (Figures 7O and S6C), which is recognized to be associated with differentiation into macrophages (Ohradanova-Repic et al., 2016). In human PBMCs, CD27 is expressed predominantly on T cells (median 60% $CD4^+$ and 91% $CD8^+$), a small subset of B cells (24%), and a far smaller number of NK cells (2%), but not monocytes (Figures S6D–S6F), thereby suggesting that downregulation of CD14 on monocytes potentially occurs indirectly via anti-huCD27 stimulation of T cells. In support of this, purified monocytes do not downregulate CD14 on anti-huCD27 stimulation (median fluorescence intensity [MFI], 7183) (Figure 7P). Re-addition of purified T cells, but not B cells, resulted in the downregulation of CD14 on monocytes.

DISCUSSION

The success of individual mAbs in cancer therapy suggests that combining them will enhance efficacy. However, it is not yet understood how best to combine these mAbs and which combinations will be effective. Our data demonstrate a way in which an immunostimulatory mAb, anti-CD27, can be used to augment the activity of a direct tumor-targeting mAb (anti-CD20) to elicit experimental cures. Anti-CD20 binds to B cells and mediates ADCP of the mAb-opsonized cells. Addition of anti-CD27 stimulates $CD8^+$ T cells and NK cells, inducing the release of CCL3, CCL4, and CCL5, which potentially attract myeloid cells.

(F) Tumor-bearing mice were treated with isotype control, anti-CD20, anti-CD27, or in combination, with or without an IFNγ-neutralizing mAb. Splenocytes were harvested on day 13, and FcγR expression was examined on macrophages. n = 6–8 per group.

(G) Naive mice were treated with an isotype control or anti-CD27, and peritoneal macrophages were harvested on day 13. Macrophages were co-cultured with carboxyfluorescein succinimidyl ester (CFSE)-labeled huCD20 tg B cells opsonized with an isotype control or anti-CD20 for 1 hr, and CFSE and F4/80 double-positive cells were examined. The phagocytic index is shown. Means are shown, n = 12.

Wilcoxon test was used to analyze p values in monocytes and neutrophils in (C) and (E–G), and Student's t test for the remaining data; $^*p < 0.05$, $^{**}p < 0.01$, $^{***}p < 0.001$, $^{****}p < 0.0001$. See also Figure S4.

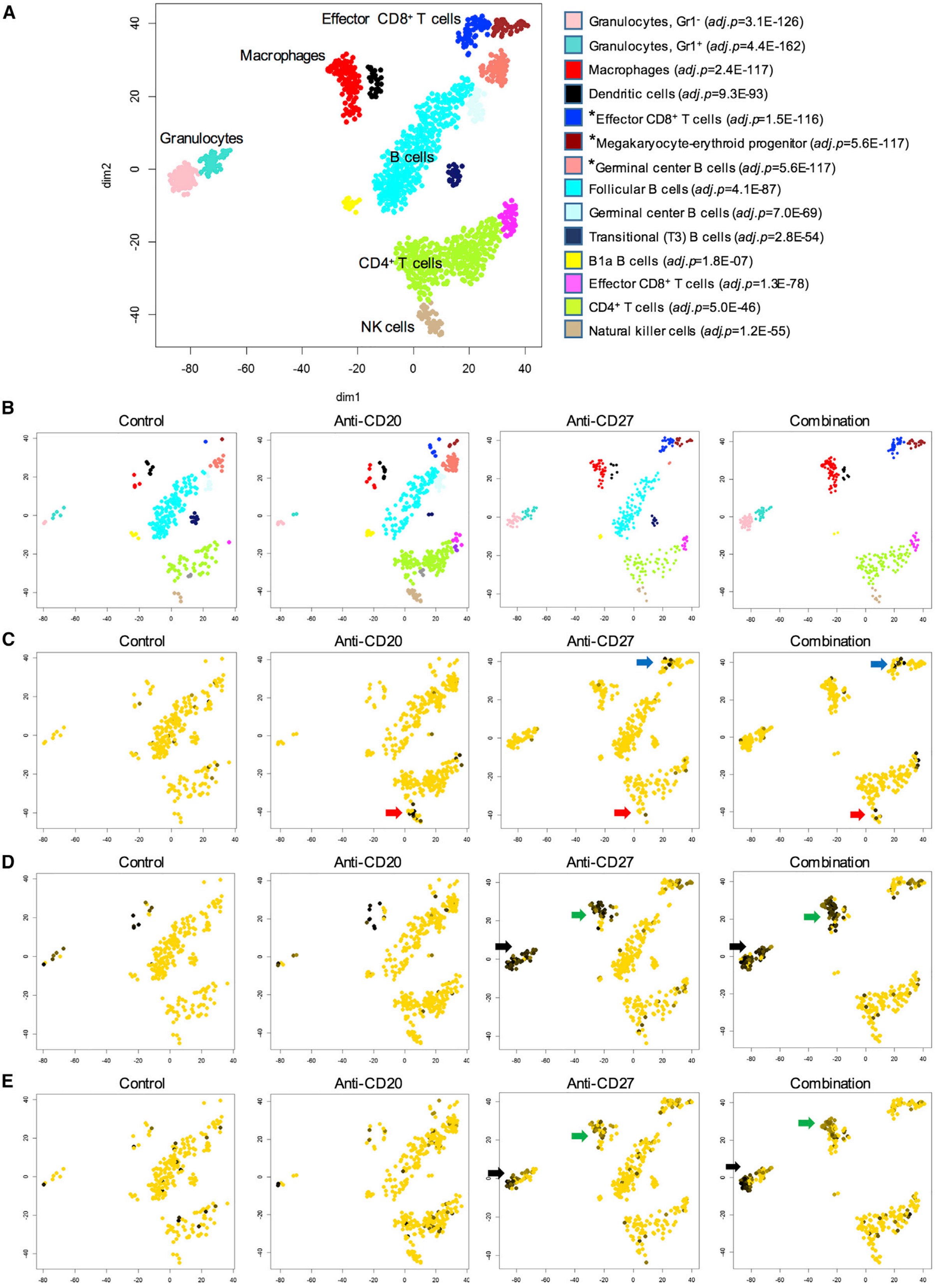

(legend on next page)

Stimulation of CD8$^+$ T cells, and to a lesser extent NK cells, by anti-CD27 also induces release of IFNγ, which activates macrophages to express more FcγRIV and promotes their inflammatory capacity, thus increasing both the number of macrophages available to perform ADCP and their phagocytic ability.

The superior therapy observed with the anti-CD20/CD27 combination could be a summation of effects as anti-CD27 on its own significantly prolongs the survival of mice in this model. Two lines of evidence indicate that this is not the case. First, the depletion of CD4$^+$ and CD8$^+$ T cells entirely abrogates anti-CD27 activity, but not combination therapy, signifying that in combination, alternative, synergistic mechanisms of anti-tumor activity come into play. Second, the 4-1BB mAb was also highly effective as monotherapy but did not result in a robust combination effect with anti-CD20 in the BCL$_1$ model.

There is ample evidence to support the role of CD27 as a costimulatory receptor on T cells. Humans deficient in CD27 (van Montfrans et al., 2012) or CD70 (Izawa et al., 2017) are at risk of lymphoproliferative disorders associated with Epstein-Barr virus (EBV) as a result of reduced proliferation of EBV-specific T cells. Our data demonstrate that anti-CD27 monotherapy is entirely dependent on T cells in the BCL$_1$ lymphoma model. Similarly, the anti-huCD27 mAb, varlilumab, is dependent on T cells for therapeutic effects in EG7 lymphoma and CT26 colon carcinoma models (He et al., 2013).

Existing literature describing the effects of CD27 stimulation on NK cells is limited (Kelly et al., 2002; Takeda et al., 2000). *In vitro* assays demonstrate that engagement of CD27 by an agonistic mAb induces proliferation and IFNγ secretion of NK cells but without any evidence of direct cytotoxicity (Takeda et al., 2000). In a B16cOVA model of anti-CD27 therapy, NK cells were shown to be essential in early tumor control but dispensable when tumor engraftment was established (Roberts et al., 2010). Here, *Ifng* upregulation was observed on both T and NK cells with CD27 stimulation by single-cell RNA sequencing, albeit the number of NK cells collected were small, thus reducing our ability to detect changes in NK cells. At the protein level, evidence of direct NK activation by anti-CD27 is supported by the upregulation of KLRG1, which is expressed on mature, fully activated NK cells (Huntington et al., 2007). In a pulmonary metastatic colorectal model, KLRG1$^+$ NK cells were shown to be crucial in protecting against tumor development in a perforin-dependent manner (Malaise et al., 2014). Altogether, these data suggest that anti-CD27 is able to directly stimulate NK cells to enhance cytokine effector function and possibly also direct cytotoxicity. Indeed, when anti-CD20 and anti-CD27 were administered in combination, depletion of both T and NK cells was required to abrogate therapy, indicating that loss of either population can in part be compensated by anti-CD27-mediated activation of the other.

We consistently observed increased infiltration of monocytes and neutrophils in naive and BCL$_1$-bearing WT and huCD27 models. Potentially, CCL3, CCL4, and CCL5 released by anti-CD27-stimulated effector CD8$^+$ T cells account for the myeloid infiltration but this needs further validation. Either a genetic model deficient for all three chemokines or simultaneous neutralization of these chemokines would be required. Given the redundancy between chemokines and their receptors as well as the numerous effector cells involved here, we hypothesize that these investigations will be complex to interpret. Nonetheless, depletion of T and NK cells effectively abrogated both myeloid cell infiltration and activation, thereby pointing to these cells as the source of myeloid chemo-attractants.

Previous investigations by many different groups have demonstrated that macrophage-mediated ADCP is the main mechanism by which anti-CD20 depletes tumor in syngeneic models (Beers et al., 2010; Gong et al., 2005; Minard-Colin et al., 2008; Uchida et al., 2004). Further, FcγRI and FcγRIV are the two critical activatory FcγRs involved in macrophage-mediated ADCP (Biburger et al., 2011; Gul et al., 2014; Hamaguchi et al., 2006; Tipton et al., 2015a). In our model, upregulation of FcγRIV on macrophages, monocytes, and neutrophils, and FcγRI on monocytes and neutrophils, initially suggests that either of these phagocytic cells or FcγR might lead to enhanced ADCP ability. Further work is required to confirm the contribution of each of these activatory FcγRs to the enhanced phagocytosis. Depletion of neutrophils had no effect on BCL$_1$ survival or *in vivo* B cell depletion ability, thus ruling out their contribution. While macrophages may be the critical immediate effectors here, the increased numbers of monocytes might also translate to increased macrophage numbers following subsequent monocyte differentiation in the tissue.

Initially contradictory to these findings are others' observations that tumor-associated macrophages are conventionally associated with poor prognosis across a number of different cancers as they have the capacity to support tumor growth through secretion of immunosuppressive cytokines among other mechanisms (reviewed by Weiskopf and Weissman, 2015). Indeed, we observed that depletion of macrophages slowed the growth of BCL$_1$. However, these associations are largely in the setting of chemotherapy as opposed to immunotherapy. In this context, it must be remembered that macrophages are also the key effectors of ADCP, and in follicular lymphoma where rituximab is employed, increased numbers of tumor-associated macrophages predict favorable outcome (Taskinen et al., 2007). Most significantly, our human *in vitro* data corroborate our murine findings by demonstrating that the anti-huCD27 mAb, varlilumab, promotes monocyte viability and potentially differentiation into macrophages. These effects occur via direct CD8$^+$ T cell stimulation as demonstrated by the lack of CD14

Figure 6. Global Gene Expression Profiling of Anti-CD20 and/or Anti-CD27 Treated BCL$_1$-Bearing Mice

(A–E) Single cell RNA sequencing was performed on spleens harvested on day 13 from BCL$_1$-bearing mice treated as described in Figure 4. t-Distributed stochastic neighbor embedding (t-SNE) plots are shown, with each point representing a cell. (A) t-SNE plot showing the individual immune effector subsets, assigned according to their top match with the coexpression atlas of the Immunological Genome Project. Each population is denoted by a different color and those marked with an asterisk (*) are proliferating. (B) Data from (A) are subdivided into the different treatment conditions to demonstrate changes in the various subsets. (C) Cells in the various condition groups expressing *Ccl3* (effector CD8$^+$ T cells and NK cells) are indicated by the blue and red arrows, respectively. The yellow cells lack expression of *Ccl3*. (D) Upregulation of *Ifitm3* on granulocytes and macrophages is indicated by the black and green arrows, respectively. (E) Upregulation of *Isg15* on granulocytes and macrophages is indicated by the black and green arrows, respectively. See also Figure S5.

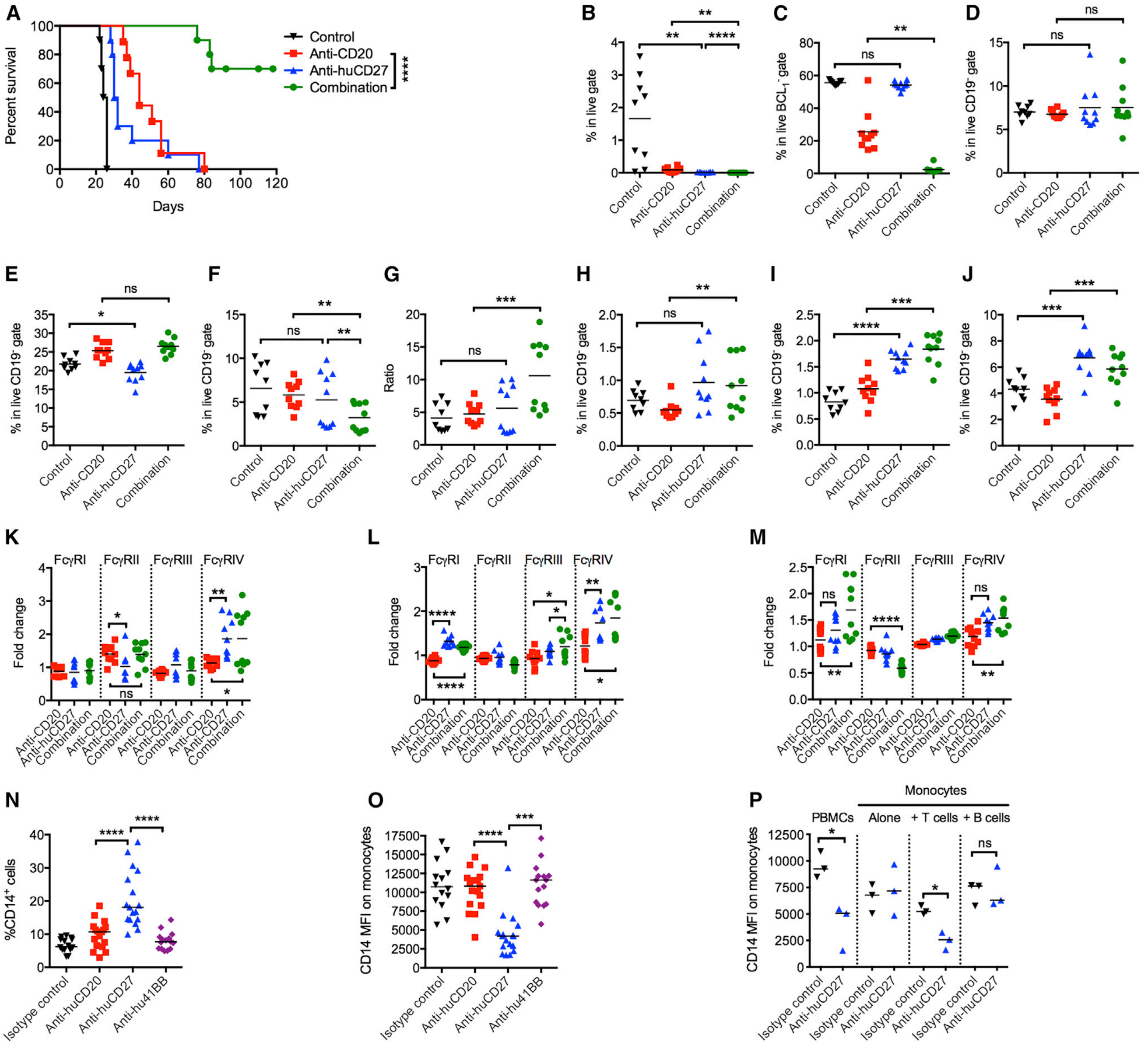

Figure 7. *In Vivo* and *In Vitro* Activity of Anti-huCD27

(A) BCL_1-bearing, transplanted huCD27 tg mice were treated as in Figure 2A and a Kaplan-Meier survival curve generated, n = 10 per group, from two independent experiments.

(B–J) BCL_1-bearing huCD27 tg mice were treated as in (A), and splenocytes were harvested on day 13 and examined for percentages of BCL_1 tumor (B), normal B cells (C), NK cells (D), $CD8^+$ T cells (E), Tregs (F), CD8/Treg ratio (G), macrophages (H), monocytes (I), and neutrophils (J). n = 4–5/group, means are shown.

(K–M) FcγR expression of macrophages (K), monocytes (L), and neutrophils (M) of mice treated in (A). n = 4–5/group, means are shown.

(N) Human PBMCs were cultured with anti-huCD20, anti-huCD27, anti-hu4-1BB, or an isotype control mAb for 48 hr. Cells were harvested and analyzed by flow cytometry. Percentage of $CD14^+$ cells are shown in the graph. Means are shown, n = 15–17 per group.

(O) The level of CD14 expression on the monocytes gated from (N) are shown. Means are shown, n = 15–17 per group.

(P) Human PBMCs, purified monocytes, purified monocytes, and purified T cells were cultured with an isotype control, or anti-huCD27 for 48 hr. The level of CD14 expression on monocytes is shown. Medians are shown, n = 3/group.

Wilcoxon test was used to assess p values for (C, F, H, L, N, and O) and Student's t test was used for the rest of the data; *$p < 0.05$, **$p < 0.01$, ***$p < 0.001$, ****$p < 0.0001$. See also Figure S6.

downregulation on monocytes with T cell depletion. It is of particular interest that an agonistic mAb to 4-1BB did not produce the same effects, despite being another co-stimulator receptor on T cells. We suspect these differences are due to the fact that CD27 is constitutively expressed, whereas 4-1BB expression requires prior activation.

Based on these data, a phase II, multicenter clinical trial has been initiated in relapsed and/or refractory B cell lymphoma to

test the combination of rituximab and varlilumab. Finally, our data indicate that anti-CD27 is also effective in enhancing direct tumor-targeting mAbs beyond anti-CD20, such as anti-gp75 in a melanoma model. We anticipate that anti-CD27 can be used to enhance the effects of other direct-targeting mAbs such as anti-CD38 in myeloma and anti-EGFR in solid tumors, and we are in the process of investigating this.

STAR★METHODS

Detailed methods are provided in the online version of this paper and include the following:

- KEY RESOURCES TABLE
- CONTACT FOR REAGENT AND RESOURCE SHARING
- EXPERIMENTAL MODEL AND SUBJECT DETAILS
  - Mice
  - Cell Lines
  - Human Samples
- METHOD DETAILS
  - Lymphocyte Isolation and Flow Cytometry
  - Tumor Models
  - *In Vivo* Cell Depletion and IFNγ Neutralization
  - *Ex Vivo* Phagocytosis Assay
  - Bone Marrow Isolation and Reconstitution of Mice
  - Antibodies
  - Cytokine and Chemokine Profiling
  - Single Cell RNA Sequencing
  - Immunohistochemistry
  - *In Vitro* Human PBMC Based Assays
- QUANTIFICATION AND STATISTICAL ANALYSIS
  - Statistical Analysis
- DATA AND SOFTWARE AVAILABILITY

SUPPLEMENTAL INFORMATION

Supplemental Information includes six figures and can be found with this article online at https://doi.org/10.1016/j.ccell.2017.11.001.

AUTHOR CONTRIBUTIONS

Conceptualization, M.J.G. and S.H.L.; Methodology, M.J.J.R.-Z., K.H., and S.H.L.; Investigation, A.H.T., K.L.C., M.J.J.R.-Z., K.H., J.T., L.N.D., H.T.C.C., S.J., V.L.F., M.J.C., H.J.K., L.-Z.H., and L.J.T.; Formal Analysis, S.M.T. and S.H.L.; Writing – Original Draft, S.H.L.; Writing – Review & Editing, T.K., A.A.-S., P.W.M.J., M.J.J.R.-Z., M.J.C., S.M.T., S.A.B., M.S.C., and M.J.G.; Resources, J.J.W.; Supervision, S.H.L.

ACKNOWLEDGMENTS

The authors wish to thank Dr. Marjolein van Egmond and Dr. Rens Braster for advice on the B16 melanoma model, and Dr. Gestur Vidarsson for the anti-gp75 antibody. Supported by Cancer Research UK clinician scientist fellowship C30010/A15269 to S.H.L., Kay Kendall Leukaemia Fund KKL 584, Wessex Medical Research Innovation Fund 2011 R06 and 2014 U10, Leuka Charity, John Goldman Fellowship 2016/JGF/0003 to M.J.J.R.-Z., and the Southampton CRUK Centre Development Fund. J.T., L.J.T., L.-Z.H., and T.K. are employees and shareholders of Celldex Therapeutics. S.H.L. is co-inventor on a patent application filed (JDM84560P.GBA) and receives research funding from Celldex Therapeutics. A.A. is co-inventor on patent applications filed (WO 2008051424 A3; JDM84560P.GBA) and receives research funding from Celldex Therapeutics. M.J.G. is inventor on patent applications filed (WO 2008051424 A3; JDM84560P.GBA). M.S.C. is co-inventor on patent application filed (JDM84560P.GBA).

Received: April 11, 2017
Revised: August 28, 2017
Accepted: October 27, 2017
Published: November 30, 2017

REFERENCES

Akiba, H., Nakano, H., Nishinaka, S., Shindo, M., Kobata, T., Atsuta, M., Morimoto, C., Ware, C.F., Malinin, N.L., Wallach, D., et al. (1998). CD27, a member of the tumor necrosis factor receptor superfamily, activates NF-kappaB and stress-activated protein kinase/c-Jun N-terminal kinase via TRAF2, TRAF5, and NF-kappaB-inducing kinase. J. Biol. Chem. *273*, 13353–13358.

al-Shamkhani, A., Birkeland, M.L., Puklavec, M., Brown, M.H., James, W., and Barclay, A.N. (1996). OX40 is differentially expressed on activated rat and mouse T cells and is the sole receptor for the OX40 ligand. Eur. J. Immunol. *26*, 1695–1699.

Arens, R., Tesselaar, K., Baars, P.A., van Schijndel, G.M., Hendriks, J., Pals, S.T., Krimpenfort, P., Borst, J., van Oers, M.H., and van Lier, R.A. (2001). Constitutive CD27/CD70 interaction induces expansion of effector-type T cells and results in IFNgamma-mediated B cell depletion. Immunity *15*, 801–812.

Beers, S.A., Chan, C.H., James, S., French, R.R., Attfield, K.E., Brennan, C.M., Ahuja, A., Shlomchik, M.J., Cragg, M.S., and Glennie, M.J. (2008). Type II (tositumomab) anti-CD20 monoclonal antibody out performs type I (rituximab-like) reagents in B-cell depletion regardless of complement activation. Blood *112*, 4170–4177.

Beers, S.A., French, R.R., Chan, H.T., Lim, S.H., Jarrett, T.C., Vidal, R.M., Wijayaweera, S.S., Dixon, S.V., Kim, H., Cox, K.L., et al. (2010). Antigenic modulation limits the efficacy of anti-CD20 antibodies: implications for antibody selection. Blood *115*, 5191–5201.

Biburger, M., Aschermann, S., Schwab, I., Lux, A., Albert, H., Danzer, H., Woigk, M., Dudziak, D., and Nimmerjahn, F. (2011). Monocyte subsets responsible for immunoglobulin G-dependent effector functions in vivo. Immunity *35*, 932–944.

Bichi, R., Shinton, S.A., Martin, E.S., Koval, A., Calin, G.A., Cesari, R., Russo, G., Hardy, R.R., and Croce, C.M. (2002). Human chronic lymphocytic leukemia modeled in mouse by targeted TCL1 expression. Proc. Natl. Acad. Sci. USA *99*, 6955–6960.

Biswas, S.K., and Mantovani, A. (2010). Macrophage plasticity and interaction with lymphocyte subsets: cancer as a paradigm. Nat. Immunol. *11*, 889–896.

Brezinsky, S.C., Chiang, G.G., Szilvasi, A., Mohan, S., Shapiro, R.I., MacLean, A., Sisk, W., and Thill, G. (2003). A simple method for enriching populations of transfected CHO cells for cells of higher specific productivity. J. Immunol. Methods *277*, 141–155.

Burris, H.A., Infante, J.A., Ansell, S.M., Nemunaitis, J.J., Weiss, G.R., Villalobos, V.M., Sikic, B.I., Taylor, M.H., Northfelt, D.W., Carson, W.E., et al. (2017). Safety and activity of varlilumab, a novel and first-in-class agonist anti-CD27 antibody, in patients with advanced solid tumors. J. Clin. Oncol. *35*, 2028–2036.

Carr, J.M., Carrasco, M.J., Thaventhiran, J.E., Bambrough, P.J., Kraman, M., Edwards, A.D., Al-Shamkhani, A., and Fearon, D.T. (2006). CD27 mediates interleukin-2-independent clonal expansion of the CD8+ T cell without effector differentiation. Proc. Natl. Acad. Sci. USA *103*, 19454–19459.

Carter, M.J., Cox, K.L., Blakemore, S.J., Turaj, A.H., Oldham, R.J., Dahal, L.N., Tannheimer, S., Forconi, F., Packham, G., and Cragg, M.S. (2016). PI3Kdelta inhibition elicits anti-leukemic effects through Bim-dependent apoptosis. Leukemia *31*, 1423–1433.

Cobb, L.M., Glennie, M.J., McBride, H.M., Breckon, G., and Richardson, T.C. (1986). Characterisation of a new murine B cell lymphoma. Br. J. Cancer *54*, 807–818.

CellPress

Dahal, L.N., Dou, L., Hussain, K., Liu, R., Earley, A., Cox, K.L., Murinello, S., Tracy, I., Forconi, F., Steele, A.J., et al. (2017). STING activation reverses lymphoma-mediated resistance to antibody immunotherapy. Cancer Res. *77*, 3619–3631.

de Jong, R., Loenen, W.A., Brouwer, M., van Emmerik, L., de Vries, E.F., Borst, J., and van Lier, R.A. (1991). Regulation of expression of CD27, a T cell-specific member of a novel family of membrane receptors. J. Immunol. *146*, 2488–2494.

French, R.R., Taraban, V.Y., Crowther, G.R., Rowley, T.F., Gray, J.C., Johnson, P.W., Tutt, A.L., Al-Shamkhani, A., and Glennie, M.J. (2007). Eradication of lymphoma by CD8 T cells following anti-CD40 monoclonal antibody therapy is critically dependent on CD27 costimulation. Blood *109*, 4810–4815.

George, A.J., McBride, H.M., Glennie, M.J., Smith, L.J., and Stevenson, F.K. (1991). Monoclonal antibodies raised against the idiotype of the murine B cell lymphoma, BCL1 act primarily with heavy chain determinants. Hybridoma *10*, 219–227.

Gill, S., Vasey, A.E., De Souza, A., Baker, J., Smith, A.T., Kohrt, H.E., Florek, M., Gibbs, K.D., Jr., Tate, K., Ritchie, D.S., and Negrin, R.S. (2012). Rapid development of exhaustion and down-regulation of eomesodermin limit the antitumor activity of adoptively transferred murine natural killer cells. Blood *119*, 5758–5768.

Gong, Q., Ou, Q., Ye, S., Lee, W.P., Cornelius, J., Diehl, L., Lin, W.Y., Hu, Z., Lu, Y., Chen, Y., et al. (2005). Importance of cellular microenvironment and circulatory dynamics in B cell immunotherapy. J. Immunol. *174*, 817–826.

Gravestein, L.A., Amsen, D., Boes, M., Calvo, C.R., Kruisbeek, A.M., and Borst, J. (1998). The TNF receptor family member CD27 signals to Jun N-terminal kinase via Traf-2. Eur. J. Immunol. *28*, 2208–2216.

Guirnalda, P., Wood, L., Goenka, R., Crespo, J., and Paterson, Y. (2013). Interferon gamma-induced intratumoral expression of CXCL9 alters the local distribution of T cells following immunotherapy with *Listeria monocytogenes*. Oncoimmunology *2*, e25752.

Gul, N., Babes, L., Siegmund, K., Korthouwer, R., Bogels, M., Braster, R., Vidarsson, G., ten Hagen, T.L., Kubes, P., and van Egmond, M. (2014). Macrophages eliminate circulating tumor cells after monoclonal antibody therapy. J. Clin. Invest. *124*, 812–823.

Hamaguchi, Y., Xiu, Y., Komura, K., Nimmerjahn, F., and Tedder, T.F. (2006). Antibody isotype-specific engagement of Fcgamma receptors regulates B lymphocyte depletion during CD20 immunotherapy. J. Exp. Med. *203*, 743–753.

He, L.Z., Prostak, N., Thomas, L.J., Vitale, L., Weidlick, J., Crocker, A., Pilsmaker, C.D., Round, S.M., Tutt, A., Glennie, M.J., et al. (2013). Agonist anti-human CD27 monoclonal antibody induces T cell activation and tumor immunity in human CD27-transgenic mice. J. Immunol. *191*, 4174–4183.

Heng, T.S., and Painter, M.W.; Immunological Genome Project Consortium (2008). The Immunological Genome Project: networks of gene expression in immune cells. Nat. Immunol. *9*, 1091–1094.

Huntington, N.D., Tabarias, H., Fairfax, K., Brady, J., Hayakawa, Y., Degli-Esposti, M.A., Smyth, M.J., Tarlinton, D.M., and Nutt, S.L. (2007). NK cell maturation and peripheral homeostasis is associated with KLRG1 up-regulation. J. Immunol. *178*, 4764–4770.

Izawa, K., Martin, E., Soudais, C., Bruneau, J., Boutboul, D., Rodriguez, R., Lenoir, C., Hislop, A.D., Besson, C., Touzot, F., et al. (2017). Inherited CD70 deficiency in humans reveals a critical role for the CD70-CD27 pathway in immunity to Epstein-Barr virus infection. J. Exp. Med. *214*, 73–89.

Keller, A.M., Schildknecht, A., Xiao, Y., van den Broek, M., and Borst, J. (2008). Expression of costimulatory ligand CD70 on steady-state dendritic cells breaks CD8+ T cell tolerance and permits effective immunity. Immunity *29*, 934–946.

Kelly, J.M., Darcy, P.K., Markby, J.L., Godfrey, D.I., Takeda, K., Yagita, H., and Smyth, M.J. (2002). Induction of tumor-specific T cell memory by NK cell-mediated tumor rejection. Nat. Immunol. *3*, 83–90.

Kohrt, H.E., Houot, R., Goldstein, M.J., Weiskopf, K., Alizadeh, A.A., Brody, J., Muller, A., Pachynski, R., Czerwinski, D., Coutre, S., et al. (2011). CD137 stimulation enhances the antilymphoma activity of anti-CD20 antibodies. Blood *117*, 2423–2432.

Loetscher, M., Gerber, B., Loetscher, P., Jones, S.A., Piali, L., Clark-Lewis, I., Baggiolini, M., and Moser, B. (1996). Chemokine receptor specific for IP10 and mig: structure, function, and expression in activated T-lymphocytes. J. Exp. Med. *184*, 963–969.

Malaise, M., Rovira, J., Renner, P., Eggenhofer, E., Sabet-Baktach, M., Lantow, M., Lang, S.A., Koehl, G.E., Farkas, S.A., Loss, M., et al. (2014). KLRG1+ NK cells protect T-bet-deficient mice from pulmonary metastatic colorectal carcinoma. J. Immunol. *192*, 1954–1961.

Minard-Colin, V., Xiu, Y., Poe, J.C., Horikawa, M., Magro, C.M., Hamaguchi, Y., Haas, K.M., and Tedder, T.F. (2008). Lymphoma depletion during CD20 immunotherapy in mice is mediated by macrophage FcgammaRI, FcgammaRIII, and FcgammaRIV. Blood *112*, 1205–1213.

Ohradanova-Repic, A., Machacek, C., Fischer, M.B., and Stockinger, H. (2016). Differentiation of human monocytes and derived subsets of macrophages and dendritic cells by the HLDA10 monoclonal antibody panel. Clin. Transl. Immunology *5*, e55.

Otten, M.A., van der Bij, G.J., Verbeek, S.J., Nimmerjahn, F., Ravetch, J.V., Beelen, R.H., van de Winkel, J.G., and van Egmond, M. (2008). Experimental antibody therapy of liver metastases reveals functional redundancy between Fc gammaRI and Fc gammaRIV. J. Immunol. *181*, 6829–6836.

Ramakrishnan, P., Wang, W., and Wallach, D. (2004). Receptor-specific signaling for both the alternative and the canonical NF-kappaB activation pathways by NF-kappaB-inducing kinase. Immunity *21*, 477–489.

Robbins, S.H., Tessmer, M.S., Mikayama, T., and Brossay, L. (2004). Expansion and contraction of the NK cell compartment in response to murine cytomegalovirus infection. J. Immunol. *173*, 259–266.

Roberts, D.J., Franklin, N.A., Kingeter, L.M., Yagita, H., Tutt, A.L., Glennie, M.J., and Bullock, T.N. (2010). Control of established melanoma by CD27 stimulation is associated with enhanced effector function and persistence, and reduced PD-1 expression of tumor infiltrating CD8(+) T cells. J. Immunother. *33*, 769–779.

Robinson, M.D., McCarthy, D.J., and Smyth, G.K. (2010). edgeR: a Bioconductor package for differential expression analysis of digital gene expression data. Bioinformatics *26*, 139–140.

Rowley, T.F., and Al-Shamkhani, A. (2004). Stimulation by soluble CD70 promotes strong primary and secondary CD8+ cytotoxic T cell responses in vivo. J. Immunol. *172*, 6039–6046.

Slavin, S., and Strober, S. (1978). Spontaneous murine B-cell leukaemia. Nature *272*, 624–626.

Souza-Fonseca-Guimaraes, F., Blake, S.J., Makkouk, A., Chester, C., Kohrt, H.E., and Smyth, M.J. (2016). Anti-CD137 enhances anti-CD20 therapy of systemic B-cell lymphoma with altered immune homeostasis but negligible toxicity. Oncoimmunology *5*, e1192740.

Sugita, K., Robertson, M.J., Torimoto, Y., Ritz, J., Schlossman, S.F., and Morimoto, C. (1992). Participation of the CD27 antigen in the regulation of IL-2-activated human natural killer cells. J. Immunol. *149*, 1199–1203.

Takeda, K., Oshima, H., Hayakawa, Y., Akiba, H., Atsuta, M., Kobata, T., Kobayashi, K., Ito, M., Yagita, H., and Okumura, K. (2000). CD27-mediated activation of murine NK cells. J. Immunol. *164*, 1741–1745.

Taraban, V.Y., Rowley, T.F., Tough, D.F., and Al-Shamkhani, A. (2006). Requirement for CD70 in CD4+ Th cell-dependent and innate receptor-mediated CD8+ T cell priming. J. Immunol. *177*, 2969–2975.

Taskinen, M., Karjalainen-Lindsberg, M.L., Nyman, H., Eerola, L.M., and Leppa, S. (2007). A high tumor-associated macrophage content predicts favorable outcome in follicular lymphoma patients treated with rituximab and cyclophosphamide-doxorubicin-vincristine-prednisone. Clin. Cancer Res. *13*, 5784–5789.

Tipton, T.R., Mockridge, C.I., French, R.R., Tutt, A.L., Cragg, M.S., and Beers, S.A. (2015a). Anti-mouse FcgammaRIV antibody 9E9 also blocks FcgammaRIII in vivo. Blood *126*, 2643–2645.

Tipton, T.R., Roghanian, A., Oldham, R.J., Carter, M.J., Cox, K.L., Mockridge, C.I., French, R.R., Dahal, L.N., Duriez, P.J., Hargreaves, P.G., et al. (2015b).

Antigenic modulation limits the effector cell mechanisms employed by type I anti-CD20 monoclonal antibodies. Blood *125*, 1901–1909.

Turaj, A.H., Dahal, L.N., Beers, S.A., Cragg, M.S., and Lim, S.H. (2017). TLR-3/9 agonists synergize with anti-ErbB2 mAb-letter. Cancer Res. *77*, 3376–3378.

Tutt, A.L., French, R.R., Illidge, T.M., Honeychurch, J., McBride, H.M., Penfold, C.A., Fearon, D.T., Parkhouse, R.M., Klaus, G.G., and Glennie, M.J. (1998). Monoclonal antibody therapy of B cell lymphoma: signaling activity on tumor cells appears more important than recruitment of effectors. J. Immunol. *161*, 3176–3185.

Uchida, J., Hamaguchi, Y., Oliver, J.A., Ravetch, J.V., Poe, J.C., Haas, K.M., and Tedder, T.F. (2004). The innate mononuclear phagocyte network depletes B lymphocytes through Fc receptor-dependent mechanisms during anti-CD20 antibody immunotherapy. J. Exp. Med. *199*, 1659–1669.

van Lier, R.A., Borst, J., Vroom, T.M., Klein, H., Van Mourik, P., Zeijlemaker, W.P., and Melief, C.J. (1987). Tissue distribution and biochemical and functional properties of Tp55 (CD27), a novel T cell differentiation antigen. J. Immunol. *139*, 1589–1596.

van Montfrans, J.M., Hoepelman, A.I., Otto, S., van Gijn, M., van de Corput, L., de Weger, R.A., Monaco-Shawver, L., Banerjee, P.P., Sanders, E.A., Jol-van der Zijde, C.M., et al. (2012). CD27 deficiency is associated with combined immunodeficiency and persistent symptomatic EBV viremia. J. Allergy Clin. Immunol. *129*, 787–793.e6.

Vitale, L.A., He, L.Z., Thomas, L.J., Widger, J., Weidlick, J., Crocker, A., O'Neill, T., Storey, J., Glennie, M.J., Grote, D.M., et al. (2012). Development of a human monoclonal antibody for potential therapy of CD27-expressing lymphoma and leukemia. Clin. Cancer Res. *18*, 3812–3821.

Weiner, G.J. (2015). Building better monoclonal antibody-based therapeutics. Nat. Rev. Cancer *15*, 361–370.

Weiskopf, K., and Weissman, I.L. (2015). Macrophages are critical effectors of antibody therapies for cancer. MAbs *7*, 303–310.

Williams, E.L., Tutt, A.L., Beers, S.A., French, R.R., Chan, C.H., Cox, K.L., Roghanian, A., Penfold, C.A., Butts, C.L., Boross, P., et al. (2013). Immunotherapy targeting inhibitory Fcgamma receptor IIB (CD32b) in the mouse is limited by monoclonal antibody consumption and receptor internalization. J. Immunol. *191*, 4130–4140.

Xiao, Y., Hendriks, J., Langerak, P., Jacobs, H., and Borst, J. (2004). CD27 is acquired by primed B cells at the centroblast stage and promotes germinal center formation. J. Immunol. *172*, 7432–7441.

Yakes, F.M., Chinratanalab, W., Ritter, C.A., King, W., Seelig, S., and Arteaga, C.L. (2002). Herceptin-induced inhibition of phosphatidylinositol-3 kinase and Akt Is required for antibody-mediated effects on p27, cyclin D1, and antitumor action. Cancer Res. *62*, 4132–4141.

STAR★METHODS

KEY RESOURCES TABLE

REAGENT or RESOURCE	SOURCE	IDENTIFIER
Antibodies		
CD3 PerCP eFluor 710 (17A2)	eBioscience	Cat. # 46-0032; RRID: AB_1834428
CD3 FITC (KT3)	In-house	N/A
CD8a FITC (53-6.7)	eBioscience	Cat. # 11-0081; RRID: AB_464914
CD44 PE (IM7)	eBioscience	Cat. # 12-0441; RRID: AB_465663
CD62L APC (MEL-14)	eBioscience	Cat. # 17-0621; RRID: AB_469409
CD4 FITC (RM4-5)	eBioscience	Cat. # 11-0042; RRID: AB_464898
CD25 APC (PC61.5)	eBioscience	Cat. # 17-0251; RRID: AB_469365
FOXP3 PE (NRRF-30)	eBioscience	Cat. # 12-4771; RRID: AB_464895
CD19 APC (1D3)	eBioscience	Cat. # 17-0193; RRID: AB_1659676
BCL1 FITC (Mc10-6A5)	In-house (French et al., 2007)	N/A
NKp46 PE (29A1.4)	eBioscience	Cat. # 12-3351; RRID: AB_996682
CD49b PerCP eFluor 810 (DX5)	eBioscience	Cat. # 46-5971; RRID: AB_11149865
CD11b e450 (M1/70)	eBioscience	Cat. # 48-0112; RRID: AB_1582237
F4/80 Alexa Fluor 647	Serotec	Cat. # MCA4971647; RRID: AB_2098198
Ly6g PE Cy7 (RB6-8C5)	eBioscience	Cat. # 25-5931; RRID: AB_469662
Ly6c APC eFluor 780 (HK1.4)	eBioscience	Cat. # 47-5932; RRID: AB_2573992
CD27 PE (AT124-1)	In-house	N/A
CD27 PerCP Cy5.5 (LG.3A10)	BD	Cat. # 563603
FcγR (2.4G2)	In-house	N/A
CD86 PE (GL1)	eBioscience	Cat. # 12-0862; RRID: AB_465767
Class II FITC (MS/114.15.2)	eBioscience	Cat. # 11-5321; RRID: AB_465231
B220 APC (RA3-6B2)	eBioscience	Cat. # 17-0452; RRID: AB_469395
CD5 PerCP Cy5.5 (53-7.3)	eBioscience	Cat. # 45-0051; RRID: AB_914332
CD8 (YTS169)	In-house	N/A
CD4 (GK1.5)	In-house	N/A
Asialo GM1	Biolegend	Cat. # 146002; RRID: AB_2462206
CD20 (18B12)	In-house (Williams et al., 2013)	N/A
CD27 (AT124-1)	In-house (French et al., 2007)	N/A
A31 (Mc39-16)	In-house (Tutt et al., 1998)	N/A
OX40 (OX86)	In-house (al-Shamkhani et al., 1996)	N/A
TIGIT (SAP45-9)	In-house	N/A
4-1BB (LOB12.3)	In-house (French et al., 2007)	N/A
PD-L1 (10F.9G2)	BioXcell	Cat. # BE0101; RRID: AB_10949073
PD-1 (RMP1-14)	BioXcell	Cat. # BE0146; RRID: AB_10949053
CTLA-4 (9D9)	BioXcell	Cat. # BE0164; RRID: AB_10949609
GITR (DTA-1)	BioXcell	Cat. # BE0063; RRID: AB_1107688
Ly6g (1A8)	BioXcell	Cat. # BE0075
IFNγ (XMG1.2)	BioXcell	Cat. # BE0055; RRID: AB_1107694
Gp75	Dr. Vidarsson, Sanquin nl (Otten et al., 2008)	N/A
HuCD27 (Varlilumab)	Celldex Therapeutics (Vitale et al., 2012)	N/A
HuEGFR (Cetuximab)	Southampton General Hospital Pharmacy	N/A

(*Continued on next page*)

Continued

REAGENT or RESOURCE	SOURCE	IDENTIFIER
HuCD20 (Rituximab)	Southampton General Hospital Pharmacy	N/A
HuCD20 (Ritux m2a)	In-house (Beers et al., 2008)	N/A
HuCD37 (WR17)	In-house (Beers et al., 2008)	N/A
FcγRI FITC (AT152-9)	In-house (Dahal et al., 2017)	N/A
FcγRII FITC (AT130-2)	In-house (Dahal et al., 2017)	N/A
FcγRIII FITC (AT154-2)	In-house (Dahal et al., 2017)	N/A
FcγRIV FITC (9E9)	In-house (Dahal et al., 2017)	N/A
HuCD14 Pacific Blue (TuK4)	eBioscience	Cat. # MHCD1428; RRID: AB_10373537
HuCD27 PE (O323)	eBioscience	Cat. # 12-0279; RRID: AB_10718236
HuCD4 Pacific Blue (OKT4)	Biolegend	Cat. # 317429; RRID: AB_1595438
HuCD8 V500 (RPA-T8)	BD Biosciences	Cat. # 560774; RRID: AB_1937325
HuCD56 APC (NKH1)	Biolegend	Cat. # 318310; RRID: AB_604106
HuCD3 PerCP (SK7)	Biolegend	Cat. # 344814; RRID: AB_10639948
HuCD19 APC-Cy7 (HIB19)	Biolegend	Cat. # 302218; RRID: AB_314248
Biological Samples		
Bone marrow cells from huCD27 tg mice	Celldex Therapeutics	HuCD27 tg
Chemicals, Peptides, and Recombinant Proteins		
Lymphoprep	Axis-Shield	Cat. # 07861
Liberase TL	Sigma Aldrich	Cat. # 5401020001
OCT embedding matrix	CellPath	Cat. # SMA-0100-00A
CTL-Test medium	CTL	Cat. # CTLT-010
Peroxidase Inhibitor	ThermoFisher	Cat. # 35000
Critical Commercial Assays		
RT^2 first strand kit	Qiagen	Cat. # 330401
RT^2 SYBR Green qPCR mastermix	Qiagen	Cat. # 330502
RT^2 profiler PCR array for mouse cytokines and chemokines	Qiagen	Cat. # 330231
HRP-conjugated anti-rat IgG polymer	Vector Laboratories	Cat. # PI-9400
Hematoxylin	Vector Laboratories	Cat. # H-3401
B cell isolation kit II, human	Miltenyi Biotec	Cat. # 130-091-151
B cell isolation kit, mouse	Miltenyi Biotec	Cat. # 130-090-862
T cell isolation kit, human	Miltenyi Biotec	Cat. # 130-096-535
Monocyte isolation kit, human	Miltenyi Biotec	Cat. # 130-091-153
Deposited Data		
Raw sequencing data	This paper	GEO: GSE_97037
Oligonucleotides		
Barcoded Bead SeqB: 5' –Bead–Linker-TTTTTTTAAGCAGTGGTATCAAC GCAGAGTACJJJJJJJJJJJJNNNNNNNN TTTTTTTTTTTTTTTTTTTTTTTTTTTTTTT-3'	ChemGene	N/A
Template_Switch_Oligo: AAGCAGTGGTATCAACG CAGAGTGAATrGrGrG	Eurogentec	N/A
SMART PCR primer: AAGCAGTGGTATCAACGCAGAGT	Eurogentec	N/A
New-P5-SMART PCR hybrid oligo: AATGATACGGC GACCACCGAGATCTACACGCCT GTCCGCGGAAG CAGTGGTATCAACGCAGAGT*A*C	Eurogentec	N/A
Custom Read 1 primer: GCCTGTCCGCGGAAGC AGTGGTATCAACGCAG AGTAC	IDT	N/A

(*Continued on next page*)

Continued		
REAGENT or RESOURCE	SOURCE	IDENTIFIER
Experimental Models: Cell Lines		
Mouse: BCL_1	In-house (French et al., 2007)	N/A
Mouse: A31	In-house (French et al., 2007)	N/A
Mouse: Eμ-TCL1	Gift from Dr. Egle, Salzburg Cancer Research Institute	N/A
Mouse: HuCD20 tg	In-house (Beers et al., 2008)	N/A
Experimental Models: Organisms/Strains		
Mouse: BALB/c	Charles River Laboratories	Strain: 028
Mouse: CBA/H	Charles River Laboratories	N/A
Mouse: HuCD27 tg: BALB/c.B6-Tg(hCD27)	Celldex Therapeutics	N/A
Mouse: C57BL/6	Charles River Laboratories	Strain: 027
Mouse: SCID	Charles River Laboratories	Strain: 236
Mouse: FcγRIII $^{-/-}$: BALB/c-*Fcgr3*$^{-/-}$	Gift from Dr. Verbeek, Leiden University Medical Center (Tipton et al., 2015a)	N/A
Software and Algorithms		
Prism	Graphpad	N/A
Cytobank	Cytobank	N/A
SABiosciences PCR Array Data Analysis Web portal	Qiagen	N/A
Seurat R package	Satija Lab	N/A
Cell B software	Olympus	N/A

CONTACT FOR REAGENT AND RESOURCE SHARING

Further information and requests for resources and reagents should be directed to and will be fulfilled by the Lead Contact, Sean H. Lim (s.h.lim@soton.ac.uk).

EXPERIMENTAL MODEL AND SUBJECT DETAILS

Mice

BALB/c, CBA/H, C57BL/6 and SCID mice were supplied by Charles River Laboratories and maintained in local facilities. FcγRIII$^{-/-}$ mice (Tipton et al., 2015a) were backcrossed over 10 generations onto the BALB/c background, and were gifted by Dr. Sjef Verbeek (Leiden University). All mice were fed regular chow, had water freely and were maintained in a conventional facility. SCID mice were maintained in a pathogen-free facility. For all experiments, age-matched (predominantly 8-12-week old) female mice were used. Animal experiments were conducted according to the UK Home Office license guidelines and approved by the University of Southampton Ethical Committee. Animals were randomly assigned into experimental groups and housed together under the same conditions. HuCD27 tg mice (He et al., 2013) on BALB/c background were maintained in Celldex animal facilities and used according to the Institutional Animal Care and Use Committees guidelines.

Cell Lines

BCL_1 (Slavin and Strober, 1978) and A31 (Cobb et al., 1986) B-cell lymphoma lines originated from female mice and were maintained by passage in BALB/c mice or CBA/H mice, respectively. The age of the source mice are not known. Eμ-TCL1 cells were derived from tumor-bearing Eμ-TCL1 tg mice, gifted from Dr. Egle (Salzburg Cancer Research Institute) following consent from C. Croce and Y. Pekarsky. The cells were sourced from a female mouse aged 204 days old at the time of sacrifice. HuCD20 tg B cells used for the *ex vivo* phagocytosis assay were derived from spleens of male and female huCD20 tg mice, approximately between 8 and 15 weeks of age (Beers et al., 2008). Cell lines have been authenticated by PCR (Eμ-TCL1) and flow cytometry (HuCD20 tg, BCL_1 and A31).

Human Samples

PBMCs were obtained from healthy, anonymized adult volunteers through Southampton National Blood Service after informed consent. As the samples were anonymized, the sex and age of the volunteers are not known. Density gradient centrifugation (Lymphoprep, Axis-Shield) performed within 4 hr. Use of human samples was approved by the East of Scotland Research Ethics Service, in accordance with the Declaration of Helsinki.

METHOD DETAILS

Lymphocyte Isolation and Flow Cytometry

For flow cytometric analysis, cells were suspended in a single cell suspension in staining buffer (PBS with 1% w/v BSA, 0.1% w/v sodium azide). An FcγR-blocking (2.4G2) 20 μg/ml was added for 10 min at RT prior to staining with specific antibodies for 15 min, RT. Red cell lysis was then performed (Invitrogen) for 2-3 min. The cells were then washed twice more and then analyzed directly on fixed with 4% paraformaldehyde before collection on the flow cytometer. The FcγR blocking step was omitted with FcγR expression was being examined. For intracellular staining, the cells were fixed and permeabilized (Ebioscience) for 30 min at RT, before washing twice more and staining for 20 min at RT. After two more washes, cells were collected on the flow cytometer. FACSCalibur or FACSCanto (all from BD Biosciences) were used and with data analyzed using Cytobank (Cytobank).

Peripheral blood and/or spleen suspensions were analyzed for $CD8^+$ T subsets (anti-CD3 PerCP eFluor 70, anti-CD8 FITC, anti-CD44 PE, anti-CD62L APC), Tregs (anti-CD3 PerCP eFluor 710, anti-CD4 FITC, anti-CD25 APC, anti-FOXP3 PE), BCL_1 tumour (anti-CD19 APC, anti-BCL_1 idiotype FITC), NK cells (anti-CD3 FITC, anti-NKp46 PE, anti-CD49b PerCP eFluor 710) and myeloid cells (anti-CD11b e450, anti-F4/80 Alexa Fluor 647, anti-Ly6g APC e780, anti-Ly6c PE Cy7) and CD27 expression (anti-human CD27 PE, anti-mouse CD27 PE).

To enumerate myeloid cells in the spleen, tissue digestion was performed using Liberase (Sigma Aldrich) after tissue harvest as per manufacturer's protocol. Briefly, harvested tissue was cut into small pieces and treated in Liberase TL for 15 min before mashing into a single cell suspension.

Tumor Models

Groups of BALB/c mice (n=5-6) received 10^4 BCL_1 or A31 cells intravenously on day 0 followed by anti-CD20 (18B12 mIgG2a, 200 μg) on day 4 and anti-CD27 (AT124-1 rIgG2a, 50-100 μg/injection) from day 5-8 by intraperitoneal injection. Alternatively, BCL_1-inoculated mice received an alternative immunomodulatory mAb from day 5 onwards as specified in the legends. For the A31 model, mAb therapy was repeated again at the same dose and sequence from day 15-18. The survival of the mice was assessed by regular splenic palpation and through normal humane endpoints. The assessor was blinded to the treatment received by the mice.

1 x 10^7 Eμ-TCL1 cells were intraperitoneally injected into groups of 6- to 8-week old female C57BL/6 mice and leukemic burden monitored by tail bleeds and CD5/B220 expression through flow cytometry as before (Carter et al., 2016). Animals were treated with anti-CD20 (250 μg) and anti-CD27 (100 μg) 1 day later when more than 10% $B220^+CD5^{int}$ lymphocytes were present in the blood. Animals were euthanized when humane end points were reached or >80% of lymphocytes were tumor cells and WBC counts >5 x 10^7/ml.

For the study of varlilumab, six-week old female BALB/c recipient mice were irradiated and reconstituted with huCD27 tg bone marrow cells. Eight to 10-weeks after bone marrow transplantation, animals were bled and huCD27 expression inspected by flow cytometry, before implantation with BCL_1.

In Vivo Cell Depletion and IFNγ Neutralization

For depletion experiments, mice were treated with YTS169 (CD8 depletion, in house), GK1.5 (CD4 depletion, in house) and asialo-GM1 (NK depletion, BioLegend) at doses of 0.5-1 mg, 1 mg and 10-20 μl per injection, every 5 days, from day -1 to day -16 i.p. For neutrophil depletion, mice were treated with 1A8 (BioXcell) at 0.5 mg/injection, every 3-4 days, from day 1 to day 13. For IFNγ neutralization, tumor-bearing mice were treated with anti-IFNγ (XMG1.2, 250 μg/injection) or an isotype control from day 4 to 13 i.p.

Ex Vivo Phagocytosis Assay

Naïve mice were treated with an isotype control or anti-CD27 50 μg i.p. from days 1 to 4. Peritoneal macrophages were harvested on day 8 by lavage with ice cold PBS. Cells were washed and FcγRIV expression was examined on the macrophages by flow cytometry using FcγRIV FITC and F4/80 A647. Macrophages were resuspended in complete RPMI and plated in 96-well flat bottom plates (5 x 10^5 cells per well, 100 μl volume) and rested for at least 6 hours. After resting, non-adherent cells were gently washed off, leaving behind macrophage for as effectors. Here, purified huCD20 tg B cells (Miltenyi Biotec) were labelled ith 5 μM CFSE and opsonized with 10 μg/ml anti-CD20 (Ritux m2a) or an isotype control (WR17 m2a) for 20 min, 4°C. The opsonized targets were then washed once and added to macrophages (2.5 x 10^5 per well, 100 μl volume) and co-cultured for 1 hr at 37°C. Subsequently, co-cultures stained with F4/80 A647 and washed before analysis by flow cytometry. Phagocytic cells were identified by double positivity for CFSE and F4/80 A647. The phagocytic index was calculated as fold change in increase in phagocytosis seen in anti-CD27 treated group compared to the isotype treated group expressed as 1.

Bone Marrow Isolation and Reconstitution of Mice

Eight- to 10-week old female huCD27 tg mice on BALB/c background were sacrificed and hind femora and tibiae were isolated and muscle and soft tissue removed. Isolated bones were trimmed at both ends and bone marrow flushed with in complete RPMI until bones were white. The collected cells were passed through a 70 μm sieve and centrifuged at 1500 rpm. Cells were resuspended in 10% v/v DMSO in fetal calf serum (FCS) and frozen in -80°C overnight before being transferred to liquid nitrogen storage until use.

Six-week old female BALB/c recipient mice were irradiated in split doses of 5 and 4 Gy, a day apart. 2-3 x 10^6/recipient bone marrow cells were thawed, washed and injected in PBS via tail veins. Recipients were housed in pathogen-free facilities and fed acid water. Eight to 10-weeks after bone marrow transplantation, animals were bled and huCD27 expression inspected by flow cytometry.

Antibodies

18B12 mIgG2a (anti-CD20) (Brezinsky et al., 2003) was produced in-house from published patented sequences. Parental 18B12 mIgG1 V regions were combined with mouse IgG2a constant regions (Williams et al., 2013). Ritux m2a (mIgG2a) was generated in the similar fashion (Beers et al., 2008). AT124-1 (anti-CD27) (French et al., 2007), Mc39-16 (anti-A31 idiotype) (Tutt et al., 1998), Mc10-6A5 (anti-BCL_1 idiotype) (George et al., 1991), OX86 (anti-OX40) (al-Shamkhani et al., 1996), SAP45-9 (anti-TIGIT), LOB12.3 (anti-4-1BB), YTS169 (anti-CD8), GK1.5 (anti-CD4) were produced from the culture supernatant of hybridoma cells or stably transfected Chinese hamster ovary cells.

Cytokine and Chemokine Profiling

The spleens of treated mice were harvested on day 6, snap frozen and RNA purified. RNA was converted to cDNA using the RT^2 first strand kit (Qiagen) and qPCR performed using RT^2 SYBR Green qPCR mastermix (Qiagen) and RT^2 profiler PCR array for mouse cytokines and chemokines (Qiagen) as per manufacturer's protocol. Data analysis was performed using the $\Delta\Delta C_T$ method and SABiosciences PCR Array Data Analysis Web portal.

Single Cell RNA Sequencing

The spleens of mice treated with isotype controls, anti-CD20 (200 μg, day 1), anti-CD27 (100 μg day 2-5) or in combination were harvested on day 13 and digested with Liberase (Sigma Aldrich). Single-cells (100 cells/ul) and barcoded mRNA-binding micro-particles (100 beads/μl) were suspended in droplets containing cell lysis buffer (~1nl; 50 cell /μl final concentration). Droplets were then broken and collected by centrifugation and subjected to cDNA synthesis (Maxima H- RTase), introducing the molecule and cell barcode to every transcript from a single cell (termed a 'STAMP'). 800 STAMPs from each condition were then selected for PCR amplification (15 cycles), library preparation (Nextera XT, Illumina) and Illumina sequencing by synthesis using a custom read 1 primer (NextSeq-500 platform; version 2 chemistry - high output setting; 20 bp read 1, 50 bp read 2 and an 8 bp index 1). Species-mixing experiments are routinely performed in our laboratory and have determined that our implementation of the Drop-Seq protocol robustly achieves single-cell encapsulation and captures transcriptomes from single-cells with high specificity (98.5% of cell encapsulation events are single-species; data not shown). Raw sequencing reads were converted to a sorted unmapped BAM file (FastqToSam, Picard bundled in Dropseq-tools v1.0) and filtered to remove all read-pairs with a barcode base quality of <10. The second read was trimmed at the 5' end to remove any TSO-adapter sequence and at the 3' end to remove polyA tails. Reads were aligned against mouse reference genome (mm10) using STAR aligner (v2.5.0a), then sorted/converted/merged to a BAM with a tag "GE" onto reads for data extraction. The DigitalExpression program (Dropseq-tools v1.0) performed digital counting (DGE) of the mRNA transcripts (unique molecular identifiers to avoid double counting reads/PCR duplicates) and created a DGE matrix (one measurement per gene per cell). Cells expressing less than 300 or more than 3,000 genes were excluded from further analysis. To normalize counts between samples, scaling factors were calculated using a trimmed mean of M-values using edgeR (Robinson et al., 2010). Cluster-specific genes were detected using the t-test algorithm in Seurat (Rahul Satija (NA). Seurat: R toolkit for single cell genomics. R package version 1.4.0.) ($p < 1 \times 10^{-3}$ was considered significant). Annotation of the resulting gene lists relative to the coexpression atlas of the Immunological Genome Project (Heng et al., 2008) was performed using the on-line ToppGene suite.

Immunohistochemistry

Spleens were harvested on day 9 and embedded in OCT (CellPath) and frozen in isopentane. Eight μm slices were air-dried overnight, fixed in 100% acetone and blocked with 2.5% normal goat serum and stained for BCL_1 cells (anti-BCL_1 idiotype), normal B cells (anti-B220), neutrophils (anti-Ly6c/Ly6g), macrophage (anti-F4/80) and monocyte (anti-CD14). Sections were treated with a peroxidase inhibitor (Pierce) for 10 min before incubation with an HRP-conjugated anti-rat IgG polymer for 30 min, followed by 3,3'-diaminobenzidine for 5 min, and counterstained with haematoxylin (all from Vector Laboratories). Images were recorded using a CXK41 inverted microscope equipped with a CC12 color camera, Plan Achromat 4 x 0.25 objective lens and Cell B software (all from Olympus).

In Vitro Human PBMC Based Assays

PBMCs were cultured using serum-free media (CTL-Test Medium, CTL) supplemented with glutamine (2 mM), pyruvate (1 mM), penicillin, and streptomycin (100 IU/mL) at 37°C in 5% CO_2. Cells were cultured in a 24-well plate at 1x10^7 cells/ml (1.5 x10^7 cells/well) and stimulated with cetuximab and rituximab (both from Southampton General Hospital oncology pharmacy), anti-4-1BB (clone 3/28, in house) or varlilumab (Celldex Therapeutics) at 5 μg/mL for 48 hr. Post culture, PBMCs were labelled with anti-CD14-Pacific Blue (Biolegend) and analyzed on the flow cytometer. To assess viability, annexin-V FITC staining was simultaneously performed.

Monocytes, T cells and B cells were purified using negative magnetic selection as per manufacturer's protocols (Miltenyi Biotec). T cells and B cells were added back monocytes in a ratio of 5:1 and 1:1, respectively. Cells were then culture as described above.

QUANTIFICATION AND STATISTICAL ANALYSIS

Statistical Analysis

Statistical analysis was performed using Graphpad Prism version 6. Normality was tested using the D'Agostino and Pearson, and Shapiro-Wilk tests, and transformed into logarithmic form to assess for log normal distribution. Subsequently, a two-tailed Student's t test was used to analyze parametric data and non-parametric data was analyzed by a Mann-Whitney test (unpaired data) or Wilcoxon test (paired data). To assess survival differences in immunotherapy experiments, Kaplan-Meier curves were produced and analyzed by log-rank testing. p values <0.05 were regarded as statistically significant.

DATA AND SOFTWARE AVAILABILITY

The accession number for the single cell RNA sequencing data reported in this paper is GEO: GSE_97037.

Cancer Cell, Volume *32*

Supplemental Information

Antibody Tumor Targeting Is Enhanced by CD27 Agonists through Myeloid Recruitment

Anna H. Turaj, Khiyam Hussain, Kerry L. Cox, Matthew J.J. Rose-Zerilli, James Testa, Lekh N. Dahal, H.T. Claude Chan, Sonya James, Vikki L. Field, Matthew J. Carter, Hyung J. Kim, Jonathan J. West, Lawrence J. Thomas, Li-Zhen He, Tibor Keler, Peter W.M. Johnson, Aymen Al-Shamkhani, Stephen M. Thirdborough, Stephen A. Beers, Mark S. Cragg, Martin J. Glennie, and Sean H. Lim

Supplemental Information

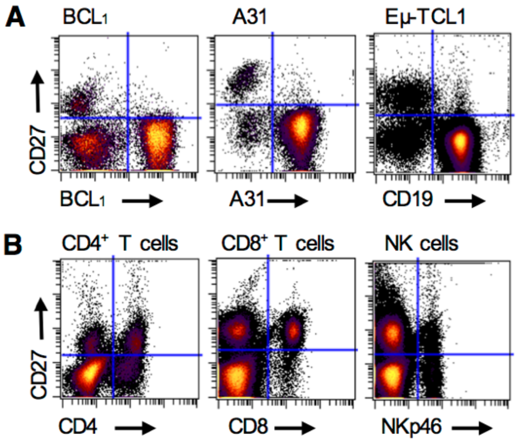

Figure S1. CD27 expression on tumor and immune effector cells. Related to Figure 2.
(A) Expression of CD27 on BCL_1, A31 and Eμ-TCL1 cells purified from mouse splenocytes are shown. Plots are representative of triplicate experiments. (B) Expression of CD27 on $CD4^+$ T cells, $CD8^+$ T cells and NK cells from mouse splenocytes are shown. Plots are representative of triplicate experiments.

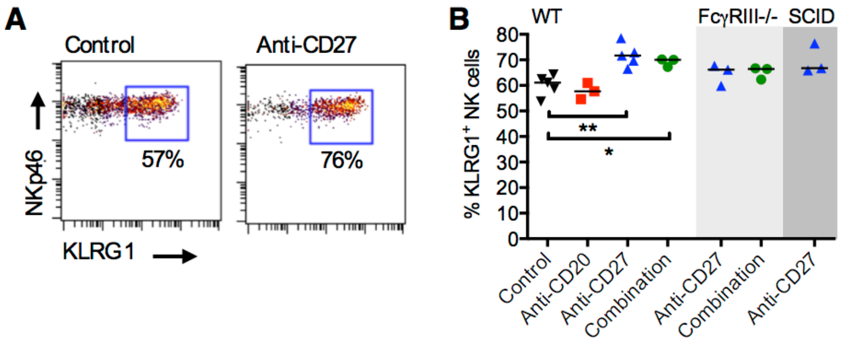

Figure S2. Anti-CD27 activation of NK cells. Related to Figure 3.
(A) Naive mice were treated with isotype control (day 1), anti-CD20 (day 1, 200 μg), anti-CD27 (day 2, 200 μg) or a combination and peripheral blood examined on day 4 for KLRG1 expression on NK cells. Representative plots are shown. **(B)** Graph shows cumulative data from (A), n=3-5. Data were assessed using Student's t test; *p<0.05, **p<0.01.

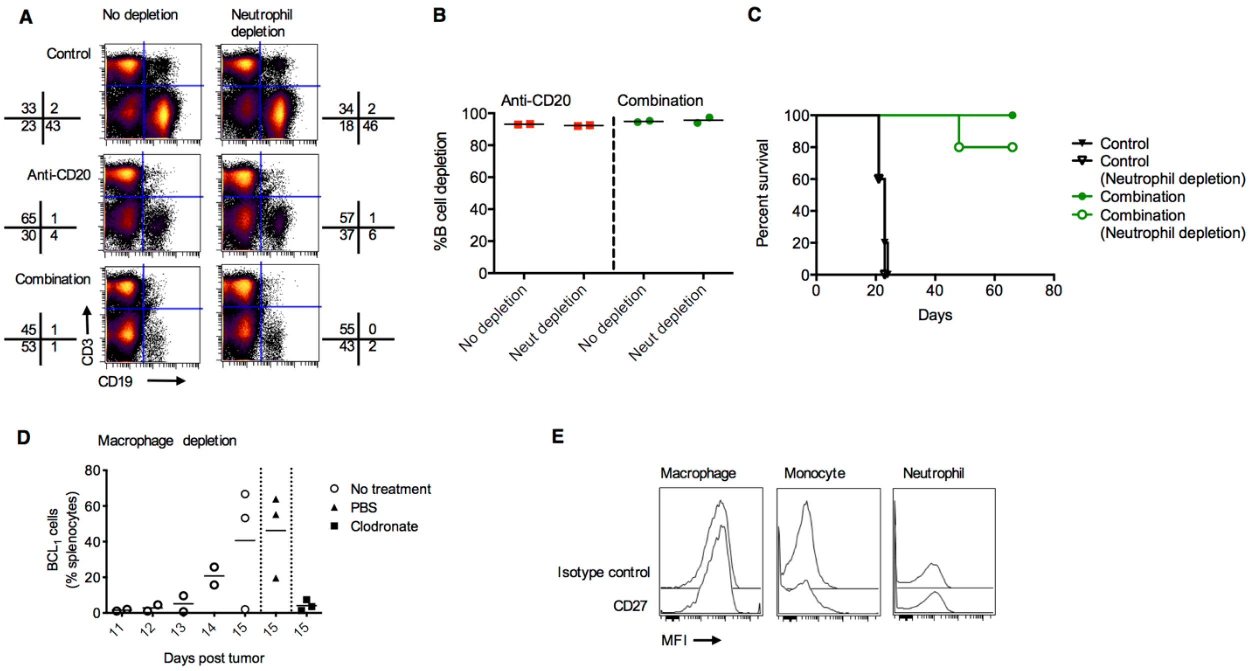

Figure S3. Role of myeloid cells in anti-CD20/CD27 therapy in BCL$_1$. Related to Figure 4.
(A) Naive mice were depleted of neutrophils and then treated with an isotype control, anti-CD20 or anti-CD20/CD27 in combination. Spleens were then harvested and the proportion of B cells in spleens assessed by flow cytometry. Representative plots are shown. (B) Cumulative data from (A), n=2 per group. (C) BCL$_1$-bearing mice were not depleted, or depleted of neutrophils and treated with either isotype control or anti-CD20/CD27 in combination. The Kaplan Meier survival curve is shown. n=9-10 per group, from two independent experiments. (D) Ten thousand BCL$_1$ cells were inoculated i.v. on day 0. Mice were then untreated or treated with PBS or clodronate liposomes on days 11 and 12, to deplete macrophages. The percentage of BCL$_1$ cells in the spleen on days 11, 12, 13, 14 and 15, post tumor inoculation is shown. (E) Expression of CD27 on macrophages, monocytes and neutrophils isolated from splenocytes of BCL$_1$-bearing mice. Histograms are representative of triplicate experiments.

A

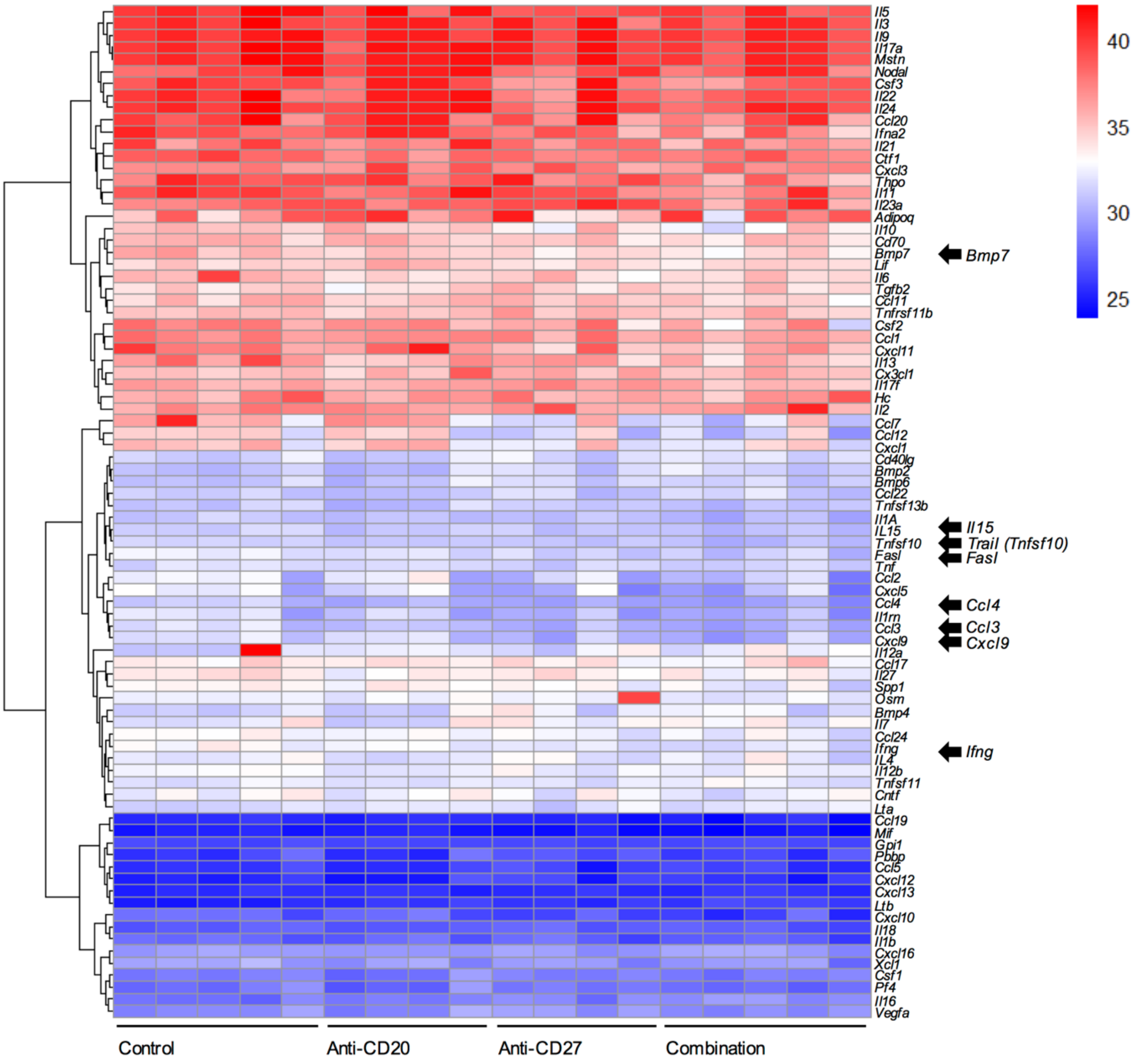
Il5
Il3
Il9
Il17a
Mstn
Nodal
Csf3
Il22
Il24
Ccl20
Ifna2
Il21
Ctf1
Cxcl3
Thpo
Il11
Il23a
Adipoq
Il10
Cd70
Bmp7
Lif
Il6
Tgfb2
Ccl11
Tnfrsf11b
Csf2
Ccl1
Cxcl11
Il13
Cx3cl1
Il17f
Hc
Il2
Ccl7
Ccl12
Cxcl1
Cd40lg
Bmp2
Bmp6
Ccl22
Tnfsf13b
Il1A
IL15
Tnfsf10
Fasl
Tnf
Ccl2
Cxcl5
Ccl4
Il1rn
Ccl3
Cxcl9
Il12a
Ccl17
Il27
Spp1
Osm
Bmp4
Il7
Ccl24
Ifng
IL4
Il12b
Tnfsf11
Cntf
Lta
Ccl19
Mif
Gpi1
Pbbp
Ccl5
Cxcl12
Cxcl13
Ltb
Cxcl10
Il18
Il1b
Cxcl16
Xcl1
Csf1
Pf4
Il16
Vegfa
Bmp7
Il15
Trail (Tnfsf10)
Fasl
Ccl4
Ccl3
Cxcl9
Ifng
40
35
30
25
Control
Anti-CD20
Anti-CD27
Combination

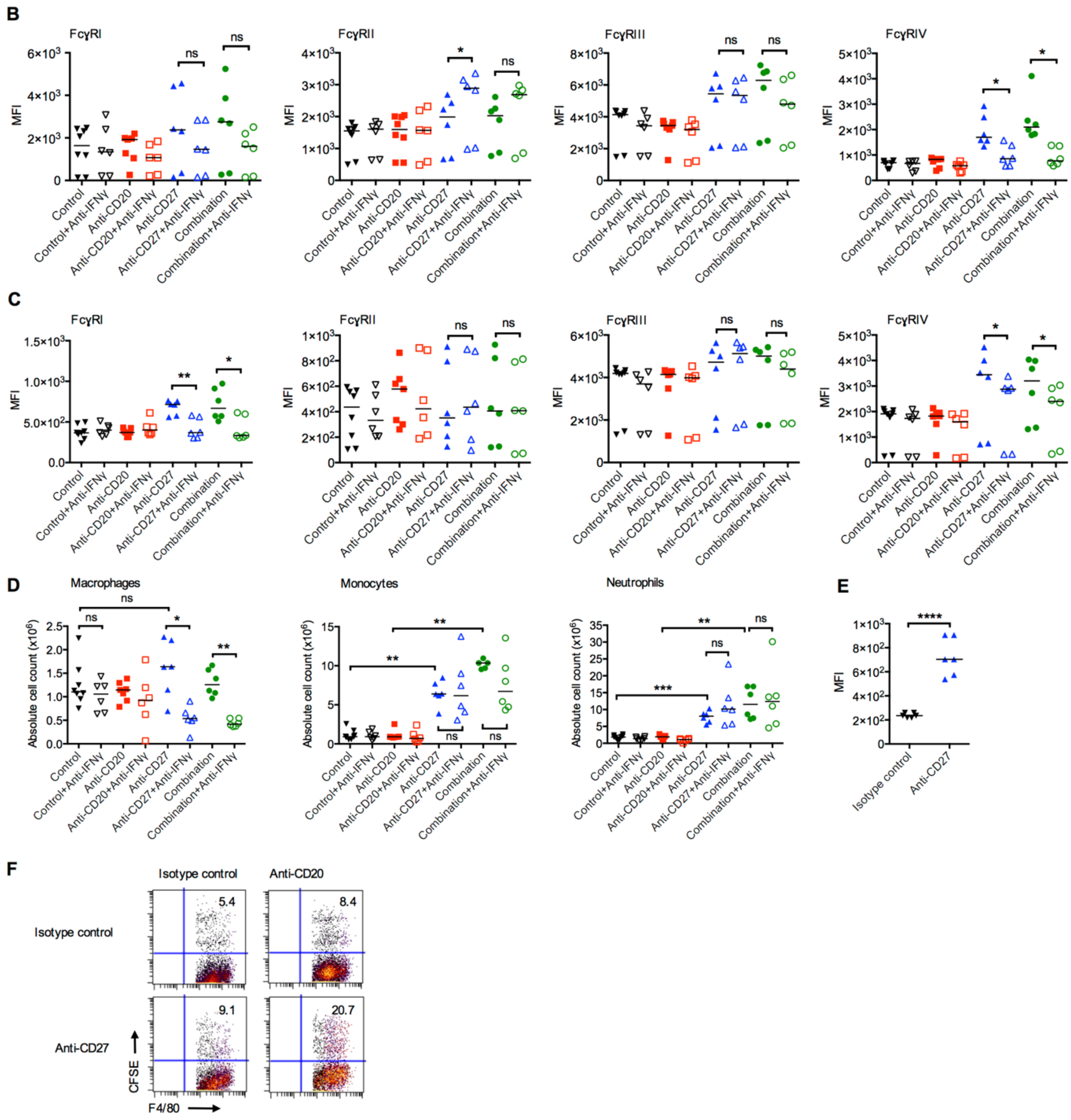

Figure S4. The effect of anti-CD27 on myeloid cells. Related to Figure 5.

(A) Naive mice were treated as described in Figure 2A. Spleens were harvested on day 9 and RNA extracted for cytokine and chemokine profiling. The heatmap shows the normalized CT values for the different treatment conditions and genes tested. (B-C) Tumor-bearing mice were treated with a neutralizing IFNγ as described in Figure 5F. Splenocytes were harvested on day 13 and FcγR expression on monocytes (B) and neutrophils (C) shown. (D) The numbers of macrophages, monocytes and neutrophils from Figure 5F were also enumerated. n=6-8 per group. (E) Naive mice were treated with an isotype control or anti-CD27 and peritoneal macrophages harvested and FcγRIV expression examined. n=6 per group. (F) Representative dot plots of the *ex vivo* phagocytosis assay from Figure 5G are shown. The double positive F4/80^{+} and CFSE^{+} cells represent phagocytic cells. The phagocytic index represents fold-increase in phagocytosis over the isotype control, which is normalized to 1. Wilcoxon test was used to assess p values in (B and C) and Student's t test for the remaining data; *p<0.05, **p<0.01, ***p<0.001, ***p<0.0001.

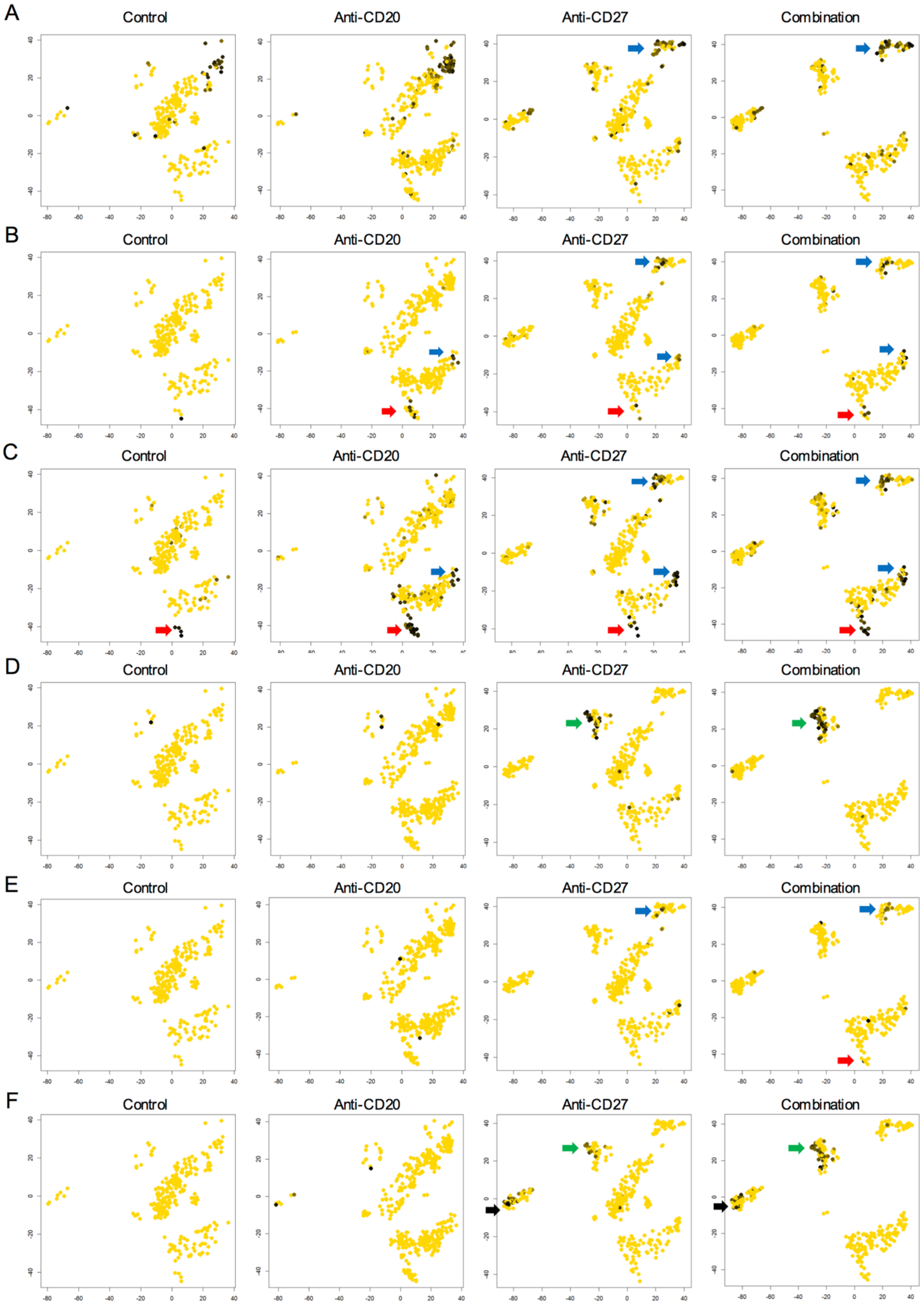

Figure S5. Single cell RNA sequencing from anti-CD20/CD27 therapy. Related to Figure 6. Mice were treated as described in Figure 6. (A) Expression of *Mki67* on effector CD8^{+} T cells are highlighted by the blue arrows. (B) *Ccl4* upregulation on effector CD8^{+} T cells (blue arrows) and NK cells (red arrows). (C)

Ccl5 upregulation is shown on NK cells (red arrows) and effector $CD8^+$ T cells (marked by blue arrows). (D) *Cxcl9* upregulation on macrophages are shown by the green arrows. (E) *Ifng* upregulation was detected on effector $CD8^+$ T cells (blue arrows), and weakly on NK cells (red arrows). (F) *Fcgr4* expression on granulocytes (black arrows) and macrophages (green arrows).

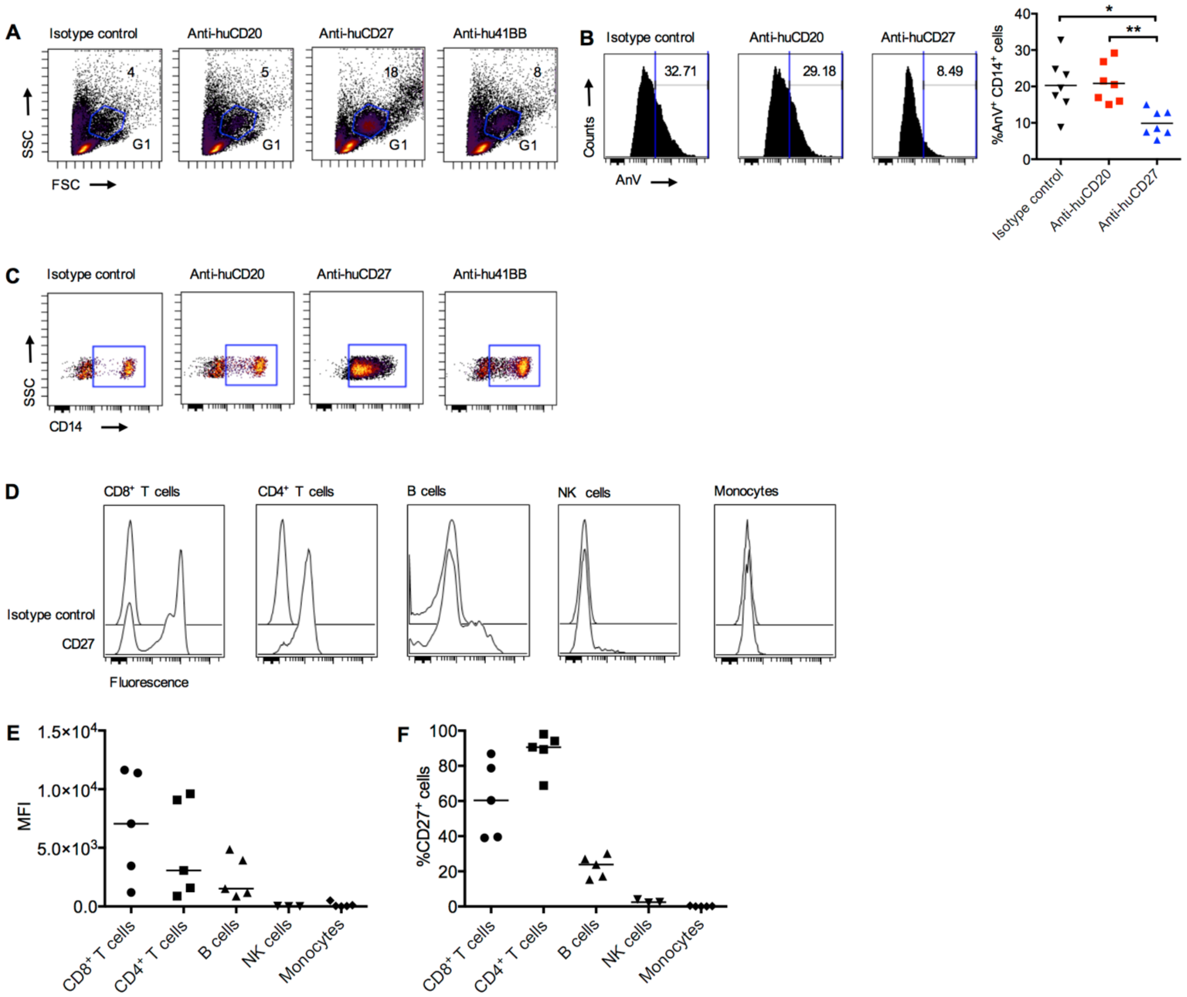

Figure S6. *In vitro* huCD27 stimulation. Related to Figure 7.

(A) Human PBMCs were treated as described in Figure 7N. The gating of monocytes by FSC and SSC are shown (G1). (B) The histograms show percentage of annexin V$^+$(AnV$^+$) gated on all CD14$^+$ cells. The cumulative % of AnV$^+$ CD14$^+$ cells are shown in the graph. n=7. p values were assessed using Student's t test; *p<0.05, **p<0.01. (C) The level of CD14 expression on G1 gated cells are shown in representative dot plots. (D) Representative histograms showing CD27 expression on CD8$^+$ and CD4$^+$ T cells, B cells, NK cells (CD56^{dim}) and monocytes. (E) MFI values from (D) are shown. (F) The % CD27$^+$ cells are shown; n=3-5 donors.
